# Assessing the vulnerability of mountain value chains to environmental and social drivers in Europe: A land-use and stakeholder-based approach

**DOI:** 10.1007/s13280-025-02153-5

**Published:** 2025-03-26

**Authors:** Pablo González-Moreno, Emilia Schmitt, Javier Moreno-Ortiz, Teresa Pinto-Correia, Nuno Guiomar, María del Mar Delgado-Serrano

**Affiliations:** 1 Research Group Evaluation and Restoration of Agroforest Systems, ERSAF, Department of Forestry Engineering, Universidad de Cordoba, Campus de Rabanales, Crta. IV, Km. 396, 14071 Córdoba, Spain; 2 ERSAF, Andalusian Institute for Earth System Research (IISTA), University of Córdoba, Campus de Rabanales, Crta. IV, Km. 396, 14071 Cordoba, Spain; 3 WEARE Research Group, Department of Agricultural Economics, Universidad de Cordoba, Campus de Rabanales, Crta. IV, Km. 396, 14071 Córdoba, Spain; 4 Departamento de Paisagem Ambiente e Ordenamento, MED – Mediterranean Institute for Agriculture, Environment and Development, CHANGE – Global Change and Sustainability Institute, Escola de Ciências e Tecnologia, Universidade de Évora, Ap. 94, 7006-554 Évora, Portugal; 5 MED – Mediterranean Institute for Agriculture, Environment and Development, CHANGE – Global Change and Sustainability Institute, Institute for Advanced Studies and Research, Universidade de Évora, Pólo da Mitra, Ap. 94, 7006-554 Évora, Portugal

**Keywords:** Adaptive capacity, Exposure, Impact, Sensitivity, Social–ecological systems

## Abstract

Mountain ranges are complex socio-ecological systems recognised as the “undervalued ecological backbone” of Europe as they provided essential ecosystem services and goods. However, we lack a deep understanding on their vulnerability to both environmental and social drivers. We carried out a stakeholder-based study assessing the vulnerability of 23 land-use systems supporting a wide range of value chains in European mountain regions. In total, 513 stakeholders participated in the evaluation of vulnerability, providing estimates for importance, exposure and sensitivity to the drivers and an assessment of 160 adaptation mechanisms. Vulnerability was calculated per region, factoring the impact of each driver and the potential reduction by adaptation mechanisms. The analysis highlighted the dominance of climate-related drivers, followed by demographic changes. Most of the adaptation mechanisms demonstrated strong social and environmental feasibility but moderate economic feasibility. Many mechanisms have shown limited implementation but offer valuable insights to reduce vulnerability in European mountain regions.

**Supplementary Information:**

The online version contains supplementary material available at 10.1007/s13280-025-02153-5.

## Introduction

Globally, around 24% of the population living in lowlands will critically depend on contributions from mountain runoff by 2050, whether for direct consumption or for food production (Viviroli et al. 2020). However, mountains are hot spots of not only water provision, but also a wider range of public goods and ecosystem services, such as food and timber production, carbon sequestration, hazard protection, mineral provision and recreation (Grêt-Regamey and Weibel 2020). In Europe, mountains cover 36% of total surface and are home to 16% of the European population (Drexler et al. 2016), crossing many national borders and providing a great diversity of ecosystems and land uses (Schröter et al. 2005). Mountain ranges are recognised as the “undervalued ecological backbone” of Europe (European Environment Agency 2010, p. 13), because the essential goods and services they provide benefit not only local communities but also are of social, economic and environmental significance for the entire continent (Egan and Price 2017; Grêt-Regamey and Weibel 2020). In fact, approximately 85M people living in European lowlands benefit from supportive mountain water contributions that flow to the Rhine–Meuse–Scheldt delta and the Danube River (Viviroli et al. 2020). The Pyrenees, the Alps, the Apennines and the Carpathians are also hot spots of wood production, soil erosion control, climate regulation, carbon sequestration and recreation (Schirpke et al. 2019; Grêt-Regamey and Weibel 2020). Furthermore, the heterogeneity imposed by extreme biophysical gradients in mountain ranges (e.g. slope and temperature) conditions land-use intensity and ecosystems driving the high compositional and functional biodiversity that can be observed across the European mountains (e.g. Nagy et al. 2012; O’Rourke et al. 2016).

Unfortunately, the decrease in the capacity to provide ecosystem services in some European mountains has already been identified by different authors (e.g. Polce et al. 2016; Grêt-Regamey and Weibel 2020). Mountain ecosystems are affected by a wide range of drivers of change different from those of the lowland areas (Kohler et al. 2010; Immerzeel et al. 2020). Particularly, they are expected to suffer far-reaching effects from global climate change (Beniston 2003), from shrinking glaciers, permafrost degradation and reduced snowpack, which significantly affect streamflow regimes and water availability for freshwater supply, hydropower, and irrigation (Beniston and Stoffel 2014; Huss et al. 2017) to increasing the frequency of extreme climatic events (Beniston 2003; Chiarle et al. 2021). Furthermore, they might also be affected by non-climatic drivers such as land-use changes (e.g. shrub encroachment due to land abandonment), soil degradation (e.g. erosion or pollution) and demographic changes (MacDonald et al. 2000; Löffler 2004; Fernandez et al. 2021).

Despite the relevance of these drivers, we have limited knowledge about how vulnerable mountain systems in Europe are, which depends not only on the intensity of the change in the driver but also on how the system perceive and can cope with it (Adger 2006). In fact, although mountain regions share the altitude relevance, they are highly diverse in terms of context and interplay of stakeholders. Thus, it is important to understand mountains vulnerability through the analytical framework of socio-ecological systems (SESs), as it allows the study of the linkages and the continuous interaction between biogeophysical and sociocultural processes that generate complex adaptive systems (Berkes et al. 2000; Alessa et al. 2018). In this context, we identify two main gaps in our knowledge of the vulnerability of European mountain systems. First, several studies have investigated vulnerability in mountain areas but focus mainly on climate change and/or single mountain ranges (e.g. Kohler et al. 2010; Sultan et al. 2022). Thus, we lack a deep understanding of the impact and consequences of the wide range of potential drivers of change that might affect the sustainability of European mountains’ socio-ecological systems. This is critical, as we expect that different mountain ranges across Europe will be affected differently by both climatic and non-climatic drivers, thereby requiring different management actions. Second, research on mountains vulnerability has predominantly focused on environmental aspects, being relatively limited the analysis of mountain areas' socio-economic vulnerability. In this regard, value chains (i.e. full range of tasks that companies and actors undertake to bring a product from conception to end-users (Crescenzi and Harman 2023)) and their associated land-use systems and local stakeholders offer a relevant SES case study across mountain regions. The vitality of rural areas depends on the existence of economic development opportunities that provide jobs and incomes to the population, the offer of social services (education, healthcare, leisure) and the access to infrastructures (transport, connectivity) and technologies. In this scenario, mountain value chains provide socio-economic opportunities to their inhabitants. They link activities located in different places, create flows of goods and services between different territories (e.g. mountains with lowlands), and generate potential leveraging (or locking-in) conditions for development (Moretti et al. 2023). The different types of agriculture, pasture and forests in mountain areas are the land-use systems that provide the key resources to the value chain (e.g. pastures and sheep cheese), at least in the first stages of production. Thus, they form the backbone of the local economy and are closely linked to other sectors in the value chain such as the food industry and tourism. Hence, the drivers of change and the vulnerability of the land-use systems have a strong influence on the maintenance of these value chains and the resilience and sustainability of the territories.

The vulnerability of complex socio-ecological systems could be analysed as the propensity of exposed elements, such as human beings, their livelihoods and assets, to suffer adverse effects when impacted by hazard events (Cardona et al. 2012). Building upon this definition, vulnerability is usually quantified with two main elements: the exposure to a hazard and the sensitivity to it (Adger 2006; Eakin and Luers 2006). Other authors include a social component, defining vulnerability as “the state of susceptibility to harm from exposure to stresses associated with environmental and social change and from the absence of capacity to adapt” (Adger 2006, p. 268). Socio-ecological systems have an intrinsic capacity to adapt and ultimately evolve to other states (Levin et al. 2013). Thus, it is fundamental to incorporate adaptive capacity in the conceptualisation of vulnerability. Here, we define adaptive capacity as the ability of a system to evolve to accommodate environmental hazards or socio-economic and policy changes and to expand the range of variability with which it can cope (Cash et al. 2006). This adaptive capacity could be mediated by specific mechanisms, defined here as human-mediated actions aimed at improving the capacity to address the vulnerability factors by adapting to or mitigating their negative effects. These actions can be management practices, norms, recommendations, strategies, plans, programmes, policies, etc. Overall, the three components of vulnerability (exposure, sensitivity and adaptive capacity) provide a comprehensive picture of a system vulnerability not only from the magnitude of the driver but also from the sensitivity to it and the capacity to adapt and cope with the change.

In this study, we propose a participatory method based on local stakeholders' perceptions to assess the vulnerability of European mountain value chains to drivers of change, based on the land-use systems where these value chains are based. Acknowledging the large differences between different mountain regions, addressing impacts on land-use systems and adopting mitigation and adaptation measures require a contextual approach and specific knowledge about stakeholders’ perceptions and attitudes (Moore and Lobell 2014). We mobilised local expert knowledge to identify vulnerability factors and drivers of change and to weigh them to identify those significantly influencing the overall vulnerability of each mountain region (Stephan et al. 2023). Specifically, the objective of this research was to (a) assess how stakeholders perceive the drivers of change affecting the vulnerability of the land-use system that support key value chain in different European mountain areas and (b) identify adaptation mechanisms that might reduce this vulnerability. We studied 23 mountain regions, covering 16 European countries that represent the wide diversity of European mountains, ranging from the highest altitudes (Alps, Carpathians, Pyrenees) to lower ones (Transdanubian, Betic Systems), including coastal and island mountains (Crete, Corsica) and national and transnational systems and a broad range of value chains (from animal-based to plant-based and tourism-based). We incorporated in the analysis a relevant range of both environmental and social drivers, building from the existing literature, on global change (e.g. Sala et al. 2000; Franklin et al. 2016) that were adapted to the local contexts.

## Materials and Methods

### Case studies, land-use systems and drivers of change

**Fig. 1:**
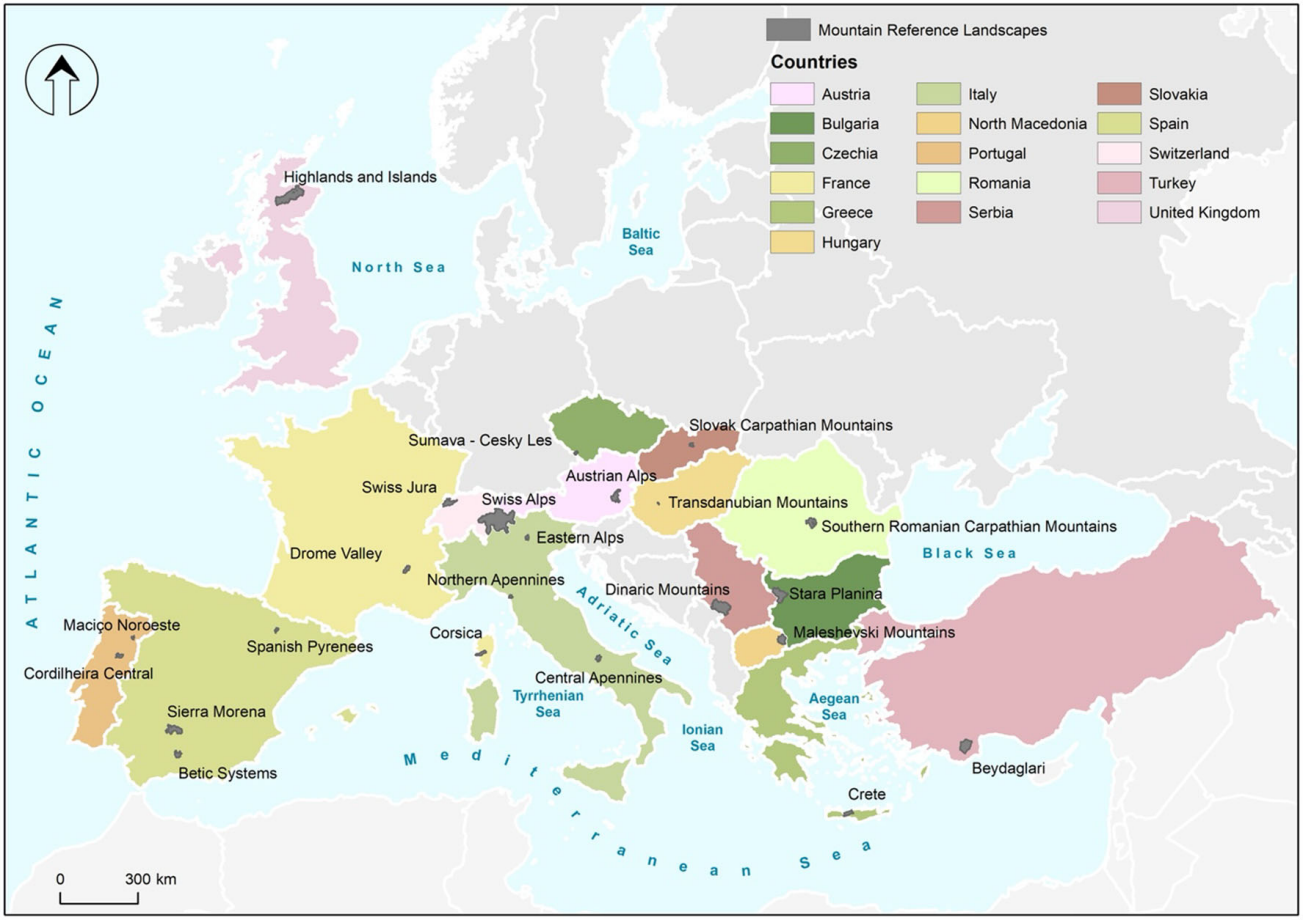
Map of the 23 study regions across 16 European countries

The analysis focused on a specific case study area within the 23 mountain regions in 16 European countries (Fig. 1), including Southern, Eastern, Central and Northern countries and EU and non-EU countries, selected for being representative of the land-use system that provides key resources to a significant value chain. The selected regions vary in size, elevation, landscape types and main land-use system (Table 1). First, in each region, a local research team collected available data and consulted a range of local stakeholders to select the most significant value chain affecting the resilience and sustainability of the mountain social–ecological system. Second, we identified the land-use system supporting each value chain and established the geographical limits of a detailed study area in each region. The land-use system definition was based on pan-European or global classifications (van Asselen and Verburg 2012; Levers et al. 2016; Malek and Verburg 2017; Rega et al. 2020).

**Table 1:** Mountain region information, land-use systems, value chains and reference variables analysed

Mountain Region	Country	Region altitude (min and max masl)	Study area (km^2^)	Land-use system	Value chain	Reference variable
Corsica	France	0–2706	836	Agroforestry system	PDO Chestnut flour	Chestnut annual production (quantity and pomological and organoleptic characteristics of the fruits)
Crete	Greece	0–773	1660	Agroforestry system	Carob flour	Carob pod annual production
Northern Apennines	Italy	50–1300	125	Agroforestry system	Chestnut flour	Chestnut annual production and tree variety influence on the quality and quantity of chestnut flour
Sierra Morena	Spain	280–726	358	Agro-silvo-pastoral system (*Dehesa*)	PDO Iberico ham "Los Pedroches"	Pasture and acorns annual production to feed the pigs
Swiss Alps	Switzerland	253–4048	378	Extensive cropland system	Grisons mountain cereal crops (wheat, rye, barley, spelt, oats and buckwheat)	Cereal yield in kg/ha
Beydaglari	Turkey	1150–2503	394	Intensive and irrigated annual crop system	Greenhouse Tomato	Greenhouse tomato annual production
Stara Planina	Bulgaria	700–1800	32	Mosaic (semi-) natural system	Public Goods from High Nature Value (HNV) farmland	Farmland biodiversity (presence of relevant habitats of European significance based on traditional agriculture)
Transdanubian Mountains	Hungary	344–709	276	Mosaic landscape	Knowledge economy in Cold Mountain Shelter	Quantity of healthy food produced through mosaic land use in agroecological farming in and around the Cold Mountain Shelter area
Maleshevski mountains	North Macedonia	660–1932	158	Mosaic landscape	Rural Tourism	Landscape of Maleshevija
Southern Romanian Carpathian Mountains	Romania	400–1000	120	Mosaic landscape	Certified Ecotourism	Landscape composition as the basis of the highly appreciated natural beauty of the area
Slovak Carpathian Mountains	Slovakia	788–895	806	Forests and grassland systems	Biohoney	Diversity of pollen- and nectar-producing plants and honeydew
Highlands and Islands	UK, Scotland	148–1281	305	Extensive rangelands	Speyside Malt Whisky	Water quantity
Sumava—Cesky Les	Czechia	250–1370	90	Permanent pastures	Beef Production	Quality pasture for cattle
Drome Valley	France	50–2453	845	Permanent pastures	Sheep meat locally produced and valorised	Grasslands and shrubbery availability on the pasture areas
Central Apennines	Italy	387–1386	2541	Agro-silvo-pastoral system	Alto Molise dairy production	Production/yield of permanent grasslands and meadows
Cordilheira Central	Portugal	496–1993	161	Permanent pastures	PDO Serra da Estrela Cheese	Pasture area available for livestock
Dinaric Mountains	Serbia	900–1300	410	Mosaic cropland (extensive) and grassland with few livestock	PDO Sjenica lamb meat	Productivity and quality of the pastures in the highlands
Swiss Jura	Switzerland	415–1606	138	Permanent pastures	PDO Tete de Moine cheese	Annual production of grass and fodder, quantity and species diversity
Austrian Alps	Austria	464–1720	1273	Permanent pastures	Lamb from the region of Weiz	Quality of pasture and links to the lamb production
Eastern Alps	Italy	181–2180	7104	Vineyards	Mountain viticulture in Alto Trentino	Vineyard productivity and grape quality
Maciço Noroeste	Portugal	108–800	753	Vineyards	Douro Wine	Vineyard productivity and grape quality
Betic Systems	Spain	500–1300	1557	Organic olive groves	Organic Mountain olive oil	Productivity of organic olive groves (quantity and quality)
Spanish Pyrenees	Spain	415–835	3414	Vineyards	Spanish Vignerons from pre-Pyrenean mountains	Vineyard productivity and grape quality

The third step was to define the link between the land-use system and the value chain by defining one reference variable per region. We define reference variable as the key element in the land-use system that characterises the main endogenous resource provided by the land-use system and is essential for the sustainability of the value chain (i.e. quality or quantity of high-altitude pasture as essential for the value chain of mountain cheese through grazing in summer). The vulnerability analysis of the land-use system is based on this variable, identifying how different drivers of change might affect it. We selected each reference variable per region based on the information gathered in the interviews (see Section “Data collection”). For most cases, the reference variable was related to productivity, the available quantity to harvest or a combination of both. In a few cases, when the resource associated with the value chain is not a productive element, other reference variables have been chosen, such as “landscape composition” or “farmland biodiversity” (Table 1).

Finally, we selected a list of ten drivers of change (Table 2) defined as any relevant natural or human-induced factors that directly or indirectly cause changes in the land-use system associated with each value chain. We began the selection with global change drivers (e.g. Sala et al. 2000; Franklin et al. 2016) that were adapted to the local contexts. For each driver of change, we listed several potential components (i.e. hazardous events) that were further redefined in each region according to the context and characteristics of the system. For example, the driver “climate change—precipitation” was characterised locally in the regions through adapted components, i.e. “decrease in rainfall in spring” in Crete, “variability in precipitation” in Hungary or simply “drought” in many other regions.

**Table 2:** General definition of drivers of change and example components to be used in the vulnerability assessment

Driver	Description and components
Climate change—precipitation	Changes in precipitation regime (rain or snow) with potential impact in the hydrological system (rivers and groundwater), soil and vegetation
Climate change—temperature	Changes in seasonal or annual mean temperature (average, maximum and minimum temperatures)
Climate change—extreme events	Changes in intensity, frequency or timing of flooding, heat waves, storms, hail and frost periods
Climate change—wildfire	Intensity, frequency or timing of wildfires
Land-use and land-cover change	Radical changes in land cover, such as conversion from forest to agriculture due to climate change or other driving forces (policies, market, etc.)
	Changes in vegetation cover such as reduction of tree cover, shrub encroachment or change in land-cover mosaic (composition and structure)
Soil physical degradation	Loss of organic matter due to erosion, poor tillage or excessive compaction
Over-exploitation of resources	Water over-extraction (surface or groundwater) Overgrazing due to livestock and wildlife density
Pests, diseases and invasive species	Changes in intensity and frequency of pest and diseases, either native or invasive
Pollution	Contamination of soil, water (surface and groundwater) or air by the discharge of harmful substances
Demographic changes	Demographic changes such as population decline or immigration-producing changes in management practices and land-use abandonment. This driver could be a cause of land-use and land-cover change

### Estimation of vulnerability

**Fig. 2:**
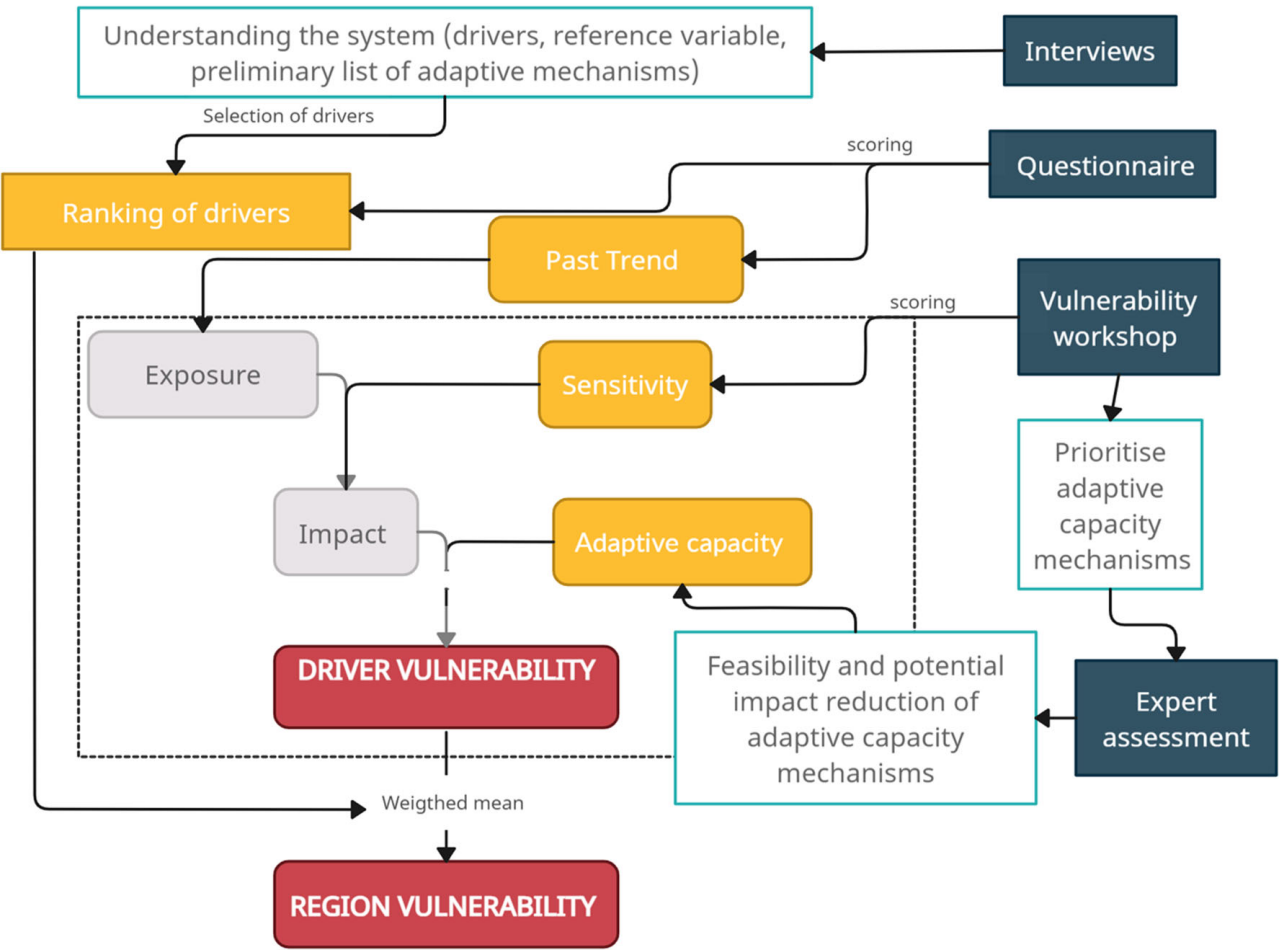
Workflow of the methodology used to assess the vulnerability to drivers of change in mountain regions. Dark blue boxes indicate participatory techniques, yellow boxes show the aspects estimated from the perception of stakeholders, grey boxes show the two components of vulnerability and red boxes show the vulnerability estimations

We implemented a comprehensive methodology to assess the future vulnerability of land-use systems to critical drivers of change in each region considering the period 2020–2040 (Fig. 2), as it is the scale most relevant to capture the perception of stakeholders at the local scale (Nissan et al. 2019). Vulnerability was estimated as a function of impact and adaptive capacity (Adger 2006; Eakin and Luers 2006) and was evaluated by stakeholders in each region in a participatory process (see following section). The impact of a driver is usually quantified with two elements: exposure (magnitude and frequency of the disturbance) and sensitivity (actual or potential degree to which a system is modified or affected by the disturbance) (Cash et al. 2006). This differentiation means that each land-use system can be affected by the driver to a different extent irrespective of the exposure. We estimated exposure based on stakeholders' perceptions of the trend of each driver in each region and land-use system in the last 20 years, regardless of whether it affects the reference variable or not. We considered this time frame to balance two aspects: (a) capture long-term trends in the drivers avoiding seasonal changes and (b) focus on a period where stakeholders could hold robust memories about past trends. We then used these perceived trends as plausible scenarios for the future target period. Here, we assume a “business-as-usual” scenario where management practices, policy and legislation remain unchanged for the target future period. We estimated sensitivity as their perception of the driver’s potential positive or negative effect on the reference variable in the next 20 years, considering the expected trends (see above). We estimated the adaptive capacity based on a set of mechanisms proposed by the stakeholders in each region, which were assessed against their feasibility and capacity to reduce the vulnerability of every driver. Finally, we quantified the current relative importance of each driver per region based on stakeholders' perception (i.e. ranking). This relative importance was used to obtain an overall value of impact and vulnerability per region.

### Data collection

The data to calculate the vulnerability to the drivers of change were gathered using different stakeholder assessment methods (i.e. interviews, questionnaires and workshops combined with expert assessment) (see the workflow in Fig. 2 and summary in Table S1). In total, 513 stakeholders participated in this study. Farmers had the highest participation rate, and men outnumbered women in all profiles (Fig. S1). However, there were more young female researchers and women in the younger age group of farmers. In the manager and extension officer profiles, the middle-aged group stood out.

#### Interviews

We carried out a preliminary assessment of the land-use systems and the potential links with the drivers of change by interviewing 10–15 relevant stakeholders representing four different profiles in each study region (total 266 across all regions): (a) farmers and producers, (b) extension officers, (c) managers and technical staff and (d) researchers. The interviews were conducted by local researchers and aimed to better understand the land-use system and the resources linked to every selected value chain. Different strategies for recruiting stakeholders (contact through the local research partners or third parties (with prior authorisation), contact with relevant actors identified by the researchers or previous contacts from other projects or activities) were combined and finally complemented with a snowball sampling strategy (Goodman 1961). From these interviews, the following information per region was obtained: (a) validation of the selected drivers and reference variable, (b) description of the specific components per driver and (c) list of adaptive capacity mechanisms linked to the drivers of change.

#### Questionnaires

With the information gathered from the interviews, we developed a questionnaire distributed online to all interviewees from the previous activity and other relevant stakeholders expected to participate in further workshops (288 respondents). These individual questionnaires provided: (a) the perceived importance of drivers based on how they currently affect the reference variable (i.e. ranking) and (b) the perceived trend for each driver of change considering the components identified. The perceived importance was assessed on a six-point scale, from 0 (it does not affect) to 5 (extremely important). The perceived trend (i.e. exposure) per driver was evaluated on a five-point scale ranging from sharp decline (−1) to sharp increase (+ 1) (Table S2).

#### Workshops

We organised a workshop in each region with a well-balanced selection of participants by profile type (262 participants, see profiles in “Interviews” section). Due to the COVID-19 situation, some workshops were held online and others in presence. The results from the previous activities were displayed to the participants by the local researchers, who moderated a follow-up discussion. The main objectives of the workshops were to: (a) validate the previous results, (b) quantify the sensitivity for each driver of change and (c) prioritise the adaptive capacity mechanisms. The sensitivity was evaluated by all participants in the workshop on a seven-class point from “high positive effect on the reference variable” (−1) to high negative effect (+ 1) (Table S3).

#### Experts’ assessment

The assessment of each adaptive mechanism in terms of feasibility and capacity to reduce vulnerability was carried out by the local researchers based on their knowledge of the system and with the support of key stakeholders. The feasibility was assessed according to economic, technical, environmental and social criteria, providing higher scores to higher feasibility levels (Table S4). The assessment of the impact reduction capacity was done per driver using four distinct groups and scores (high reduction: 0; moderate reduction: 0.6; low reduction: 0.3; and no reduction: 1). This scoring system assumes that those mechanisms with high reduction capacity can reduce the vulnerability up to the minimum level. Furthermore, we also evaluated the current implementation of the mechanism (none, few farms, several farms and most farms) and the potential agents required to implement it (producer, cooperative, professional organisations, researchers, regional government, local government, central government and EU).

### Vulnerability matrix calculation per region

Vulnerability values per region were calculated based on the aggregation of data from the participatory process according to the following steps:

1. Estimate the arithmetic mean of “ranking”, “trend” and “sensitivity” values of all individual responses per region and driver component.
2. Calculate the “impact” as the average of the mean “trend” (proxy of exposure) and mean “sensitivity” based on the rules established in MacDonald et al. (2000) (Table 3). In few cases, trend scores were estimated for more than one component per driver but just one sensitivity value. For these cases, the overall impact was calculated using the maximum trend score. Based on these calculations, impact scores range from + 1 (maximum negative value) to −1 (maximum positive value), and 0 indicates no relevant impact in the reference variable.
3. Calculate “vulnerability” per driver as the impact multiplied by the maximum reduction capacity score across all adaptive mechanisms. We applied this calculation considering a different set of adaptive mechanisms based on their feasibility scores: (a) all mechanisms, (b) only mechanisms with average feasibility higher than 2 (moderate feasibility) and (c) only mechanisms with average feasibility higher than 2.5 scores (high feasibility).
4. Overall impact and vulnerability per region were calculated as the weighted mean of all driver components considering the importance given by the “ranking”. Ranking scores were normalised (0–1) per region and driver as the mean ranking score divided by the sum of all ranking scores per region. Thus, the total calculation of vulnerability per region was estimated according to this formula:
  $${\text{Vulnerability}} = \mathop \sum \limits_{i}^{n} \left( { \frac{T + S}{2} \times A } \right) \times R$$

where *i* is each driver, *T* is the mean trend score, *S* is the mean sensitivity score, *A* is the adaptive mechanisms' reduction capacity and *R* is the mean ranking score.

**Table 3:** Impact scoring calculation considering trend and sensitivity aspects

Trend	Sensitivity	Impact calculation	Rationality
Increase (positive score)	Negative effect (positive score)	Arithmetic mean	Both trend and sensitivity increase vulnerability
Increase (positive score)	Positive effect (negative score)	Arithmetic mean. Trend transformed as negative score	Both trend and sensitivity reduce vulnerability as the driver shows a positive effect on the reference variable
Decrease (negative score)	Negative effect (positive score)	Arithmetic mean	Trend reduces vulnerability while sensitivity increases vulnerability. Both components counteract

Finally, to compare across regions, each driver component per region was linked to a generic driver classification (Table 2). When two or more components per region were related to the same generic driver, the maximum value of each component was retained. This procedure allowed us to identify the component contributing more to vulnerability per driver and region.

## Results

### Ranking, trends and sensitivity of drivers across Europe

**Fig. 3:**
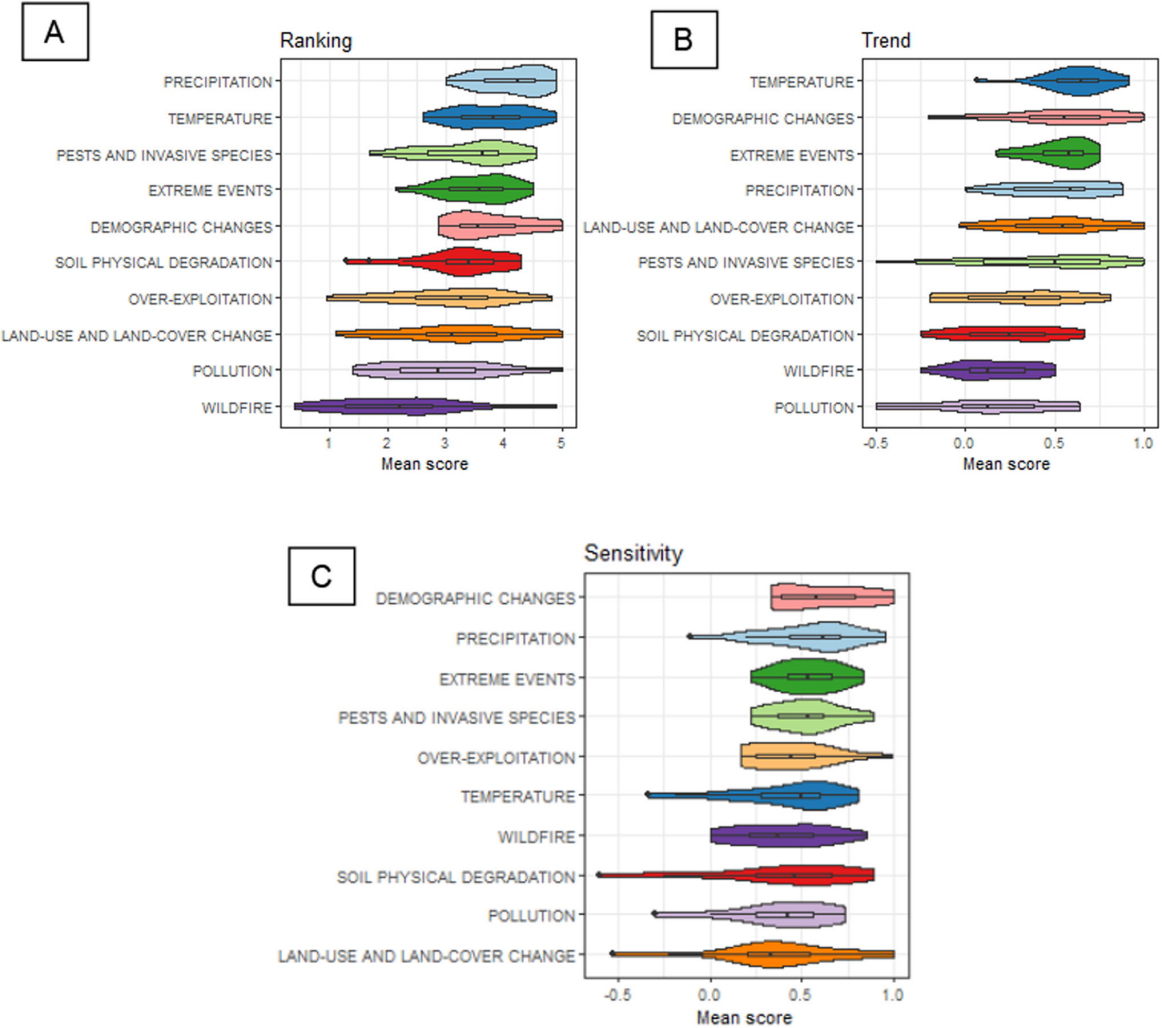
Boxplots summarising the scores of ranking (**A**), trends (**B**) and sensitivity (**C**) of the drivers across all 23 regions. The ranking score considers very low (0) to very high importance (5) while trend score shows high decrease (−1) to high increase (1). Sensitivity scores consider from high positive effect of the driver (−1) to high negative effect (1). For details in the score categories, see the Methods section. For each vulnerability component, drivers are sorted by higher mean value from top to bottom

According to the stakeholders’ perception, the highest ranked drivers of vulnerability across the 23 regions were climatic drivers, particularly precipitation and temperature, and also “extreme events” (Fig. 3A). Among non-climatic drivers, “pest and invasive species” was also relevant. Other non-climatic drivers showed high variability in importance across regions, particularly over-exploitation and land-use and land-cover change. Finally, wildfire showed the lower mean relevance across all regions, although the region of Cordilheira Central in Portugal indicated this driver as extremely highly relevant.

Stakeholders perceived an increase in magnitude in all drivers (i.e. the trend shows a mean score positive) during the last 20 years across all 23 regions studied (Fig. 3B). Particularly, temperature showed the highest increase in magnitude while pollution showed the slightest increase. Climatic drivers showed, in general, a higher increase in magnitude than other types. Some drivers presented high variability across regions indicating a high context-dependent effect. This pattern is particularly relevant for the “pest and invasive species” driver, where we have regions across all the gradient of options (i.e. −0,5 in the northern Apennines region and 1 in the Drome (France), but many regions evaluated it at 0 or not even evaluate it because of little relevance). Two other drivers, extreme events and temperature, show less variability and increasing trends across all regions (Fig. 3B). The driver precipitation displays an overall increase in magnitude, reflecting the widespread perception that the precipitation regime is vastly changing with an overall rainfall reduction across most regions.

In contrast to previous components of vulnerability, demographic changes showed the highest sensitivity across all 23 regions (Fig. 3C). Interestingly, while temperature currently showed a high relevance (high ranking score), it might show moderate sensitivity in the next 20 years, indicating that based on stakeholders’ perception, it might not affect the management practices in the medium term. In contrast, other drivers, such as precipitation changes (mostly reduction), showed high levels of importance for both current and future periods. In several regions, stakeholders perceived that some drivers such as temperature, land-use and land-cover change, and soil physical degradation might positively affect the respective land-use system. Specifically, an increase in temperature might increase productivity in some regions with moderate-temperature ranges (e.g. the Alps) or allow the crop to thrive at higher altitudes (e.g. wine production in the Pyrenees and the Italian Alps) and have the opposite effect in already warm areas (e.g. Mediterranean mountains). Land-use and land-cover change might positively affect the reference variable if the driver tends to increase the area dedicated to these land-use systems (e.g. agroecological practices are widespread). Similar rationality is found for the positive effects of the soil physical degradation driver, suggesting positive feedback between the driver and the land-use system (e.g. organic farming promoting permanent grass cover).

### Adaptive mechanisms

**Fig. 4:**
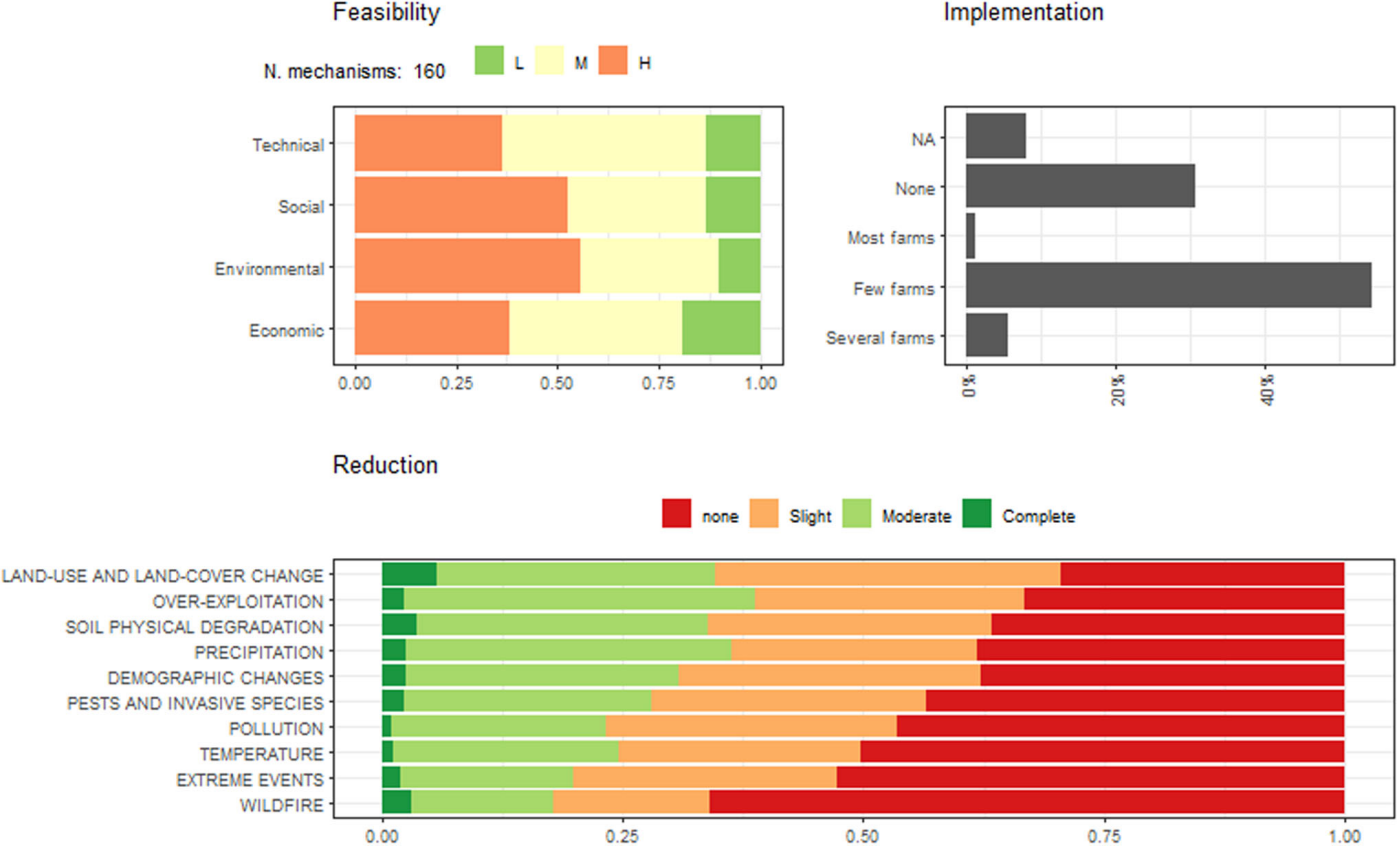
Summary of adaptive mechanisms across 23 mountain regions. Top left indicates the proportion of mechanisms in each feasibility level (L—low, M—medium and H—high) per criteria. Top right shows the proportions of mechanisms in each implementation level. Bottom plot indicates the proportion of mechanisms reducing the vulnerability across drivers of change, where complete indicates full reduction and none, absence of reduction capacity

Stakeholders from all regions suggested a total of 160 mechanisms of adaptation. These mechanisms had high social and environmental feasibility but moderate technical and economic feasibility (Fig. 4). Around 50% of these mechanisms were already in practice in some farms or communities within the regions, and approximately 30% of the mechanisms were innovative and previously unimplemented. Across drivers, non-climatic ones showed greater potential for reduction through adaptive mechanisms (assuming full implementation), particularly those linked to the land-use and land-cover change drivers (Fig. 4). However, wildfire exhibited the lowest potential for reduction across all regions. Finally, only a few mechanisms seem able to fully reduce driver vulnerability, mostly associated with the driver land-use and land-cover change.

Several mechanisms were highlighted as key to reducing the vulnerability of the system across most drivers in each region (Table S5). These mechanisms showed high diversity, including integrative practices, dissemination, research and governance. Specifically, the top four mechanisms showed a mean value reduction of 2 (maximum value of three) corresponding to four different regions. Interestingly these mechanisms were related to specific research on the predictions of productivity related to changes in climate (Swiss Jura and Crete) and the dissemination of agroecological practices and services (Swiss Alps and Betic Systems).

There were 13 adaptive mechanisms with high levels of feasibility in all four criteria (i.e. economic, technical, environmental and social; Table S6). However, these mechanisms are implemented either by just a few farms or not yet. Ten mechanisms are feasible to implement at the farm level and include, for example, replacing old orchards for adapted varieties to drought, using permaculture or shrub management. Seven of the top mechanisms with the highest feasibility would require the support or action of the EU, specifically, three of them mentioning the Common Agriculture Policy and the others related to increase knowledge transfer and support for farm improvement and adaptation (e.g. irrigation, landscape and shrub management and new varieties). Considering all adaptive mechanisms, the average feasibility scores criteria were rather similar (from 2.22 to 2.50; Table S7). The “environmental benefit” criteria had the highest average scores while “economic viability” was the minimum. Across the different organisations responsible of implementation, the average feasibility was even more similar (2.30 to 2.39), with researchers showing the lower feasibility and EU the highest (Table S7).

### Vulnerability matrices

The vulnerability analysis yielded a wide range of impact and vulnerability scores across all 23 regions (Table S4, Supplementary material 2). Six regions showed a high impact level (> 0.5) covering a wide geographical area from West to East Mediterranean (Table 4). Interestingly, few Mediterranean regions seem relatively resistant to the drivers of change studied here (e.g. Betic Systems and Crete). In general, regions covering alpine and central European ranges showed moderate to low impact of the drivers.

**Table 4:** Overall impact and vulnerability per region. Values closer to 1 indicate higher impact and vulnerability. Three different adaptation scenarios are considered to estimate vulnerability: A. with all adaptation mechanisms applied, B. only applying mechanisms with medium feasibility or higher and C. only applying mechanisms with high feasibility

Region	Impact	Vulnerability considering scenarios of adaptation		
		A. All mechanisms	B. Medium-feasibility mechanisms	C. High-feasibility mechanisms
Beydaglari	0.7	0.2	0.2	0.3
Stara Planina	0.6	0.2	0.2	0.2
Cordilheira Central	0.6	0.4	0.4	0.4
Sierra Morena	0.6	0.2	0.3	0.3
Central Apennines	0.6	0.2	0.2	0.3
South Carpatians	0.6	0.1	0.1	0.1
Drôme Valley	0.5	0.4	0.4	0.5
Jura	0.5	0.2	0.2	0.2
Pyrenees	0.5	0.1	0.2	0.3
Crete	0.5	0.1	0.1	0.1
Maleshevski mountains	0.5	0.1	0.1	0.1
Slovak Carpathians	0.5	0.2	0.2	0.3
Austrian Alps	0.4	0.1	0.1	0.3
Transdanubian mountains	0.4	0.1	0.1	0.1
Maciço Noroeste	0.4	0.1	0.1	0.2
Northern Apennines	0.4	0.2	0.2	0.3
Eastern Alps	0.4	0.1	0.1	0.2
Speyside	0.3	0.1	0.1	*
Corsica	0.3	0.0	0.0	0.0
Betic systems	0.3	0.0	0.0	0.0
Šumava—Český les	0.2	0.1	0.1	0.1
Dinaric mountains	0.2	0.1	0.1	0.1
Swiss Alps	0.1	0.0	0.0	0.0

*No mechanisms with high feasibility

Vulnerability scores account for the capacity of adaptive mechanisms to reduce the impact of the drivers. Assuming the implementation of all the adaptive mechanisms suggested per region (scenario A in Table 4), the vulnerability of the systems was significantly reduced (up to 0.5). In fact, after the reduction, the vulnerability across all regions was somewhat similar. The different adaptation scenarios (i.e. applying only the adaptation mechanisms with medium or high feasibility) showed similar results in vulnerability.

## Discussion

This study represents the most comprehensive analysis of the vulnerability of mountain land-use systems in Europe based on stakeholder perceptions. Through active engagement of more than 500 stakeholders across 23 mountain regions in Europe, we have managed to obtain the perception of farmers, managers, extension officers and researchers on the relevance of drivers of change shaping their vulnerability. We found similarities across European mountains with some drivers, such as temperature and precipitation scoring high in the ranking of most regions and demographic changes as their main driver according to the sensitivity, confirming the high relevance of climatic factors in mountain areas (Beniston 2003; Schneiderbauer et al. 2021). Despite these general patterns, we also found wide variability in impact and vulnerability across regions, reflecting a clear context-dependent effect on the vulnerability of the mountain land-use systems.

### Main drivers of vulnerability according to stakeholder perceptions

Precipitation, extreme weather events and demographic changes emerge as those with the higher influence on the vulnerability of the reference variables (i.e. sensitivity). Precipitation stands out as the primary driver in the rankings of 13 regions, spread out from south to northern Europe. Due to climate change, this driver is likely to increasingly affect the growth of crops and grassland (Fitton et al. 2019) and the health of forests (Kutnar et al. 2021). Many studies confirm that the effects of climate change will be most impactful through changes in the water cycle (Franklin et al. 2016; Arora 2019), which are impacting both highland and lowland landscapes and the capacity of mountain value chains to function. However, precipitation changes are difficult to predict and highly variable between regions (Olesen et al. 2011; Fitton et al. 2019; Immerzeel et al. 2020). Indeed, in some regions of this study, the driver was described as a change in the seasonality of rainfall, or in the form of precipitation as rain or snow, or sometimes as an increase, causing negative effects on the reference variable through excessive water. These differences in the direction of the drivers were highly challenging to synthesise into a single score for the driver. Still, collecting stakeholders’ perceptions to define the components allowed flexibility and illustration of these differences and the results aligned with changing precipitation patterns simulated by models for mountain areas (Palazzi et al. 2019).

Extreme events’ driver closely intertwines with precipitation patterns, and difficulties in differentiating both were identified in the process. Cited extreme events in the components to define this driver were extreme precipitations (very heavy rains, hail or snow), frost, storms and extreme droughts. Several regions that selected this driver mentioned that the timing of occurrence is especially crucial as, for example, extreme weather events in winter have almost no effect, but late frost or hail in spring can hamper crop growth. The shift in seasons due to climate warming is also reported in the alpine region, and farmers have already started to adapt by shifting planting cycles (Olesen et al. 2011). For instance, this effect is very relevant for pasture systems. Stakeholders in regions with pasture systems noted that drought and/or hail affect the quality and quantity of grass fodder. Similar results on the high negative impact of droughts on the productivity of grasslands have been reported for other regions (Ghahramani et al. 2019; Emadodin et al. 2021). Stakeholders also agreed that the frequency and damage from extreme events are increasing, as reflected in their high trend and sensitivity score (top 3 in Fig. 4). Similar results were also found by Czarnecki et al. (2023).

Temperature emerged as the most significant driver with the highest "trend" score, reflecting stakeholders' perception of substantial change over the last 20 years—either through increased summer days or milder winters. This trend is expected to persist and intensify. The literature confirms that warming is expected to happen faster at higher altitudes (Pepin et al. 2022). The vulnerability associated with rising temperatures notably impacts the vegetation cycle, especially during spring when early re-vegetation faces heightened susceptibility to late frosts (Moriondo et al. 2013; Lhotka and Brönnimann 2020; Marquis et al. 2022) but also the ice melting and water availability. Rising temperatures will enable crop shifts at higher altitudes, i.e. from pasture to grapes (Cardell et al. 2019), but such crops at these heights become more susceptible to late frost, rainy summers, premature snowfall, drought and unpredictable weather fluctuations (Moriondo et al. 2013). Changes in temperature and precipitation also influence the spread of pests and diseases (Cohen et al. 2020). Although stakeholders evaluated this driver with a lower impact on average, some stakeholders ranked it higher. Interestingly, the higher values were attributed in France to a sheep herding value chain, and the “pest” considered was the wolf. Wolf occurrence is mainly driven by conservation efforts, but climate change might also extend its suitable area in mountains (Reshamwala et al. 2022) exacerbating the conflicts between human activities and big predators (Kuijper et al. 2019).

While ranking lower compared to precipitation and temperature, demographic changes remained significantly ranked as the top driver in Crete and within the top three in Eastern and Mediterranean countries (Bulgaria, Romania, Portugal, Czechia, Spain and Italy). In fact, according to the sensitivity scoring, this driver showed on average the highest negative effect on the land-use systems. This finding suggests that despite stakeholders perceive climatic drivers as key elements on their systems (i.e. higher ranking), the higher negative influence is caused eventually by demographic changes (i.e. higher sensitivity). Other studies found that migration from rural regions in Europe is the main driver of land-use changes, land abandonment and decreased production (Meyer and Früh-Müller 2020; Pazúr et al. 2020). This phenomenon is driven by a complex interplay of biophysical, agricultural socio-economic and regional factors, particularly impacting agricultural land decrease, especially in mountainous areas (Pazúr et al. 2020; Perpiña Castillo et al. 2021). The projected abandonment of more than 5.6 million hectares of land by 2030 in the EU and the UK further underscores this trend (Perpiña Castillo et al. 2021). This trend can, in turn, have significant impacts on other drivers, mainly landscape composition, afforestation and soil physical properties (Nadal-Romero et al. 2023), wildfire risks (Damianidis et al. 2021), disease spread and biodiversity (Pérez-Luque et al. 2021).

The lower end of the ranking consistently includes wildfire, pollution and soil physical degradation, indicating a relatively reduced consideration compared to other drivers for the examined regions and selected reference variables. The relatively lower importance given to “wildfires” (ranked last) was not expected as at least the Mediterranean regions unveil a growing concern for wildfires (Damianidis et al. 2021). One explanation to this finding might be the relatively lower representation of forestry systems within the studied land-use systems. The exception is Cordilheira Central in Portugal that evaluated wildfires as the top driver. This region has a forest–pasture mosaic with intense abandonment trends and recent devastating fires and casualties (Castellnou et al. 2018; Rodrigues et al. 2022). Other forest landscapes, such as Sierra Morena (Spain), have the opposite pattern with high tree mortality rates deriving in lower fire risk. However, studies also highlight that stakeholders' perception differs from what science predicts regarding wildfires increase in Europe (Fernandez-Anez et al. 2021).

### Variability among the analysed regions

The most vulnerable regions, according to the overall indicator considering impact and vulnerability, are located in Turkey, Bulgaria, Portugal, Spain, Italy and Romania (see Table 4). Conversely, the least impacted are in Switzerland, Serbia and Czechia. However, generalised results and overarching regional tendencies cannot be inferred from these outcomes; regions within the same countries or nearby have important differences in the impact scores. For instance, in the Iberian Peninsula there is wide variability, with both Sierra Morena and Cordillheira Central with high impact and the Betic systems in the lower part of the ranking. Despite showing similar climate and macroeconomic context, it seems there are further differences at local level that make a major effect on how stakeholders perceive the vulnerability of their land-use systems. Several sources also emphasise the high variability in climate change impacts upon regions and land-use systems (Olesen et al. 2011; Nissan et al. 2019). Likewise, socio-economic drivers like demographic changes also display high variability in the studied regions. Overall, differences in vulnerability are substantial across regions and may be closely linked to specific contexts and the influence of drivers within the value chain. Accordingly, recommendations and analyses should be tailored to the regions and specific land-use systems.

### Influence of the adaptation mechanisms to reduce vulnerability

The collected adaptive mechanisms in this study offer pathways to enhance resilience in mountain areas facing drivers (Wyss et al. 2022). We found that only few mechanisms were able to reduce significantly the impact across the wide range of drivers that each region is facing (Table S5). In contrast, a combination of mechanisms seems to be more effective to reduce overall vulnerability. We tested two adaptation scenarios per region with different combinations of mechanisms to calculate vulnerability scores. Interestingly, applying only the adaptation mechanisms with medium or high feasibility showed similar results in vulnerability than applying all mechanisms. This finding indicates that just implementing the highly feasible mechanisms might significantly reduce the impact of the drivers with less effort and resources. After applying the mechanisms, three regions even reached zero scores of vulnerability (i.e. Corsia, Betic systems and Swiss Alps), which can be explained by both the low impact perceived in the regions and the high perception of the impact reduction capacity of the mechanisms suggested. As there are very few studies on stakeholders’ perceptions of vulnerability focused on European multiactor groups (Soubry et al. 2020), it is impossible to confirm the latter.

The 160 adaptation mechanisms identified are the first of their kind collected from stakeholders in Europe. Our findings highlight the importance of administrations, especially the EU, to support research and development of adaptation mechanisms designed on the basis of local context and stakeholder perception and the merging of scientific and local knowledge. These mechanisms in general have a higher acceptance and feasibility. However, further research is needed to determine their actual potential. For example, agroforestry was cited in a few regions as having complete or moderate reduction potential for some drivers but not against wildfires, but a recent study (Damianidis et al. 2021) showed that agroforestry is a sustainable land-use option to reduce wildfires risks in the Mediterranean region.

### Learning and challenges from the participatory process

Our research represents a novel approach as it involves a participatory analysis of stakeholders’ perceptions on the vulnerability of the land-use system that supports their livelihood (i.e. value chain). Different authors recognise the individual’s perception of risks as crucial in addressing vulnerability and taking action (Mileti 1999; Fuchs 2009; Sultan et al. 2022). This research has directly gathered knowledge from stakeholders who possess a practical and experience-based understanding of land-use systems, making their insights highly valuable (Newig et al. 2023). Knowledge and concern about risks and impacts, as well as the level of awareness of the existence of and responsibility for, are strong predictors of the attitude to adopt adaptation measures (Li et al. 2017; Mitter et al. 2019). Thus, understanding stakeholders’ perception is essential in promoting on-site adaptation and designing effective policy strategies to support them. However, the research faces a major challenge as the results heavily rely on participants' opinions, and perceptions can be dynamic, varying across individuals and groups (Mileti 1999). To mitigate this variability, the study identified relevant types of stakeholders and calculated the average of their scores. Despite the complexity of involving numerous research partners and stakeholders, the outcomes hold greater significance due to their grounded perspectives and contextualised approach, able to support decisions targeted to boost changes that reduce vulnerability.

To enhance the participatory process, we propose key actions: mapping the appropriate stakeholders for the context and topic, finding efficient ways to engage them (such as creating trustful and secure environments and sharing power), maintaining stakeholder interest and involvement throughout the process, and communicating effectively with diverse stakeholder groups, such as recommended by Newig et al. (2023). Clear communication was particularly important in the research, as it involved translating abstract terms and technical information into national and laypeople's languages and ensuring that the knowledge shared was accessible and explicit for a wide range of stakeholders. Researchers have taken significant steps to convert technical knowledge into accessible formats, such as indicators and maps, making it understandable for a diverse range of stakeholders. This effort led to a fruitful information exchange process, resulting in regional-specific knowledge that could be used to prioritise drivers of change and adaptive mechanisms. Therefore, this knowledge might be useful to effectively tackle both present and future challenges (Armitage et al. 2011). Moreover, this working method has facilitated a dialogue among stakeholders that did not exist before, leading to the creation of networks and interactions among the participants.

## Conclusion

The cross-comparison of vulnerability based on stakeholders’ perspectives across 23 mountain regions that widely represent European mountain reality provided significant results that could guide decision-makers in addressing the problems facing these regions. First, we found similarities across European mountains with some drivers, such as temperature and precipitation scoring high in the ranking of most regions and demographic changes as their main driver according to the sensitivity. This finding underscores the high perceived importance of climatic drivers but that the overall negative impact is caused by socio-economic challenges. Despite these general patterns, we also found wide variability in impact and vulnerability across regions, even within the same country, reflecting a clear context-dependent effect on the vulnerability of the mountain land-use systems that could be linked to specific characteristics of the land-use system and its local environment. Secondly, local knowledge and the feasibility to implement adaptation mechanisms emerge as crucial in addressing vulnerability in mountain regions across Europe. The high number of proposed adaptation mechanisms reflects participants’ concerns the mountain’s vulnerability and their commitment to the area’s future. Nonetheless, implementing numerous mechanisms may surpass these actors' capacities. Fortunately, just implementing high-feasibility mechanisms yielded a substantial reduction in vulnerability. This pattern highlights the importance of supporting research and developing context-specific adaptation strategies, hybridising scientific and local knowledge. Finally, building capacities and enhancing local autonomous adaptive capacity require collective efforts with administrations, especially at the European and national levels.

## Supplementary Information

Below is the link to the electronic supplementary material.

Supplementary file1 (PDF 190 KB)

Supplementary file2 (PDF 590 KB)

## Data Availability

The data that support the findings of this study are available from University of Cordoba, but restrictions apply to the availability of these data and so are not publicly available. The data are, however, available from the authors upon reasonable request.
