# Supplementary material for: Assessing the vulnerability of mountain value chains to environmental and social drivers in Europe: A land-use and stakeholder-based approach
Source: Ambio. 2025 Mar 26;54(8):1386–403. doi: 10.1007/s13280-025-02153-5 (PMC12214118; doi:10.1007/s13280-025-02153-5)
Supplement: Supplementary file 1 — Supplementary file1 (PDF 190 KB) [file 13280_2025_2153_MOESM1_ESM.pdf]

Supplementary Material 1.

Assessing the vulnerability of mountain value chains to environmental and social drivers in Europe: a land-use and stakeholder-based approach

Pablo González-Moreno[1,2*], Emilia Schmitt[3], Javier Moreno-Ortiz[3], Teresa Pinto-Correia[4], Nuno Guiomar[5], MOVING Consortium[6], María Mar Delgado-Serrano[3]

Table S1: Summary of the participatory data collection methods summarizing the objective, target group, outcomes and method for each step of

Participatory method	Interviews	Questionnaire	Workshop
**Objectives**	- Validation of the reference variable<br>- Identify key resources of LUS and their attributes<br>- Inquiry about drivers of change<br>- Additional drivers suggested<br>- Identify relevant components affecting the reference variable<br>- Identify possible underlying causes<br>- Identification of adaptive capacity mechanisms linked to drivers of change	- Feedback on driver description and components for indicators.<br>- Ranking of driver importance based on current impact on the reference variable.<br>- Perception of past trend and future projection of drivers.	- Identify the sensitivity for each driver of change<br>- Develop and prioritise the adaptive capacity mechanisms.
**Method/technique**	- Identification of other actors through snowball sampling method<br>- Open-question interview following template	- questionnaire in google forms or other tool, based on the templates provided to score<br>- ranking and trends evaluated on six-class scales	- Individual questionnaire on the sensitivity of drivers on a 7-class scale<br>- Prioritization of adaptive mechanisms by facilitation and consensus building
**Outputs**	- Initial list of drivers obtained<br>- Characterization of interviewee profiles<br>- Information recorded in an Excel template for post-processing<br>- initial list of adaptive capacity mechanisms for workshop	- Final results matrix with components, trends, and ranking values.<br>- Summary factsheet for each driver, including description, components, underlying causes, and relationships.<br>- Ranking of drivers, excluding those of low importance<br>- Actors' perception on past trends of the drivers.	- Evaluation and discussion of sensitivity of the reference variable to drivers of change<br>- List of adaptation mechanisms

the participatory process. The targe stakeholders included a diverse range of actors representing various backgrounds including farmers/producers with decision-making authority, extension officers/advisors providing technical guidance, resource managers, and researchers, ensuring a comprehensive understanding of the Land Use System and its associated value chain.

Table S2: Trend categories and numeric score associated.

Response	score
It has declined sharply in the last 20 years	-1
It has declined slightly in the last 20 years	-0.5
Constant in the last 20 years	0
It has increased slightly in the last 20 years	0.5
It has increased sharply in the last 20 years	1

Table S3: Sensitivity categories and the numeric score associated.

Response	Score
High positive effect on the reference variable. Improves to a large extent creating a large increase in profitability.	-1
Moderate positive effects on the reference variable. Improves to a medium extent creating a moderate increase in profitability.	-0.6
Limited positive effects on the reference variable. Improve the reference variable occasionally (only some years) or generally but to a small extent.	-0.3
No effect on the reference variable	0
Limited negative effects on the reference variable: require minor changes in management practices.	0.3
Moderate negative effects on the reference variable: require important changes in management practices. Affecting profitability.	0.6
High negative effects on the reference variable: will require a change in the land use system (e.g., species). The exploitation will not be profitable.	1

Table S4: Criteria for feasibility analysis of adaptation capacity mechanisms

**Numeric score**	**Economic viability**	**Technical viability**	**Environmental benefits**	**Social acceptability**
**3**	High (own resources)	High (own resources)	High (potential for biodiversity and / or ecosystem services)	High (broader positive impact to society and social support)
**2**	Medium (need local support or small credit)	Medium (need local technical support)	Medium (does not affect biodiversity or services)	Medium (no positive impact or low social opposition)
**1**	Low (need for subsidies or great credit)	Low (need for external consulting)	Low (negatively impact biodiversity and / or services)	Low (medium to strong opposition by society)

Table S5: Top List of adaptive mechanisms with higher mean capacity of reducing vulnerability across all study cases (mean reduction capacity >=1.8) including its mean feasibility (from 1 to 3) across economic, technical, social and environmental criteria.

REGION	Mechanism	Mean feasibility	Mean reduction
**Swiss Alps**	Advanced soil practices - Further sensibilisation on soil preservation and intercropping, goes together with climate mitigation strategies	3.0	2.0
**Swiss Jura**	Better use of milk potential in function of the time of year	2.8	2.0
**Betic Systems**	Dissemination of the benefits associated with the eco-systemic services provided by the ecological mountain olive grove at environmental, economic and cultural level	2.5	2.0
**Crete**	Research to improve the predictions on the effects of climate change and its impacts on the different crops in the region	2.0	2.0
**Betic Systems**	Specific informative actions on the benefits of organic production in the mountain olive grove encouraging the change of model. Specific training actions to increase the added value to the practices that producers, service companies, cooperatives, etc. carry on, contributing to the creation of a professional sector that is trained and adapted to the management needs of this particular agro-system.	2.3	1.9
**Swiss Alps**	Water capacity - New water storage solutions for summer	1.5	1.8
**Crete**	Adequacy of policies and regulations regarding agro-forestry land use systems	1.8	1.8
**Crete**	Communal collaboration to transfer traditional knowledge carob cultivation and increase awareness on responsive strategies and adaptation	2.3	1.8
**Betic Systems**	Raise awareness and disseminate good practices linked to organic mountain olive groves, generating added value for them, via premium price based on ecosystem services. Promote specific quality labels within the European Union framework.	2.0	1.8
**Betic Systems**	Increase CAP aids for organic mountain olive groves, including specific modernisation plans and the allocation of additional agri-environmental schemes for the most fragile olive groves (based on biodiversity conservation, slopes, etc.).	3.0	1.8
**Speyside**	Distillery water management to reduce consumption	2.3	1.8

Table S6: Top list of adaptive mechanisms with higher feasibility (maximum value across all criteria) across all study cases.

List of mechanisms	Current implementation	Producer	Cooperative	professional organizations	Researchers	regional government	local government	central government	EU	Region
Increase knowledge transfer	Few farms	YES	YES	NO	YES	YES	NO	YES	YES	IT-NorthApennines
Sufficient water irrigation	NA	NO	NO	NO	YES	YES	YES	YES	YES	MK-Maleshevski
Landscape and shrub management, with support from	None	YES	YES	YES	NO	YES	YES	YES	YES	PT-CordillheiraCentral
Strict (and simplified) regulations enforcement with penalties in domains related with touristic activities (i.e. building development, waste management, ATVs usage)	NA	NO	NO	NO	NO	YES	YES	YES	NO	RO-SouthCarpatians
Developing a destination management unit, to efficiently manage the eco-destination	Few farms	YES	YES	YES	YES	YES	YES	NO	NO	RO-SouthCarpatians
Investing in the quality of education in the region, in order to contribute to enhancing the attractiveness of the region for future generations	NA	NO	NO	NO	YES	YES	YES	YES	NO	RO-SouthCarpatians
Increase in CAP aid for organic mountain olive groves, including specific modernisation plans and the allocation of aid complementary to agri-environmental aid for the most fragile olive groves (biodiversity, slopes, etc.).	Few farms	YES	YES	YES	YES	YES	NO	YES	YES	ES-Betic

Mechanism 2: Advanced soil practices - Further Sensibilisation on soil preservation and intercropping, goes together with climate mitigation strategies	Few farms	YES	YES	YES	YES	YES	YES	YES	NO	CH-Alps
Increased eligibility of semi-natural grasslands for CAP Pillar 1 payments	Few farms	YES	YES	NO	NO	NO	NO	YES	YES	BG-StaraPlanina
Improved targeting of CAP Pillar 2 payments	Few farms	YES	YES	YES	NO	NO	NO	YES	YES	BG-StaraPlanina
Improved governance of common grazing	Few farms	YES	YES	YES	NO	YES	YES	YES	NO	BG-StaraPlanina
replacement old orchard by varieties adapted (drought)	Few farms	YES	NA	YES	YES	YES	YES	NO	YES	FR-Corsica
PERMACULTURE DESIGN	Few farms	YES	YES	NO	YES	NO	NO	NO	NO	HU-Transdanubian

Table S7: Average feasibility values per organization across all adaptation mechanisms

	Economic viability	Technical viability	Environmental Benefit	Social acceptability	Feasibility average
**Producer**	2.23	2.23	2.48	2.36	2.33
**Cooperative**	2.21	2.25	2.59	2.45	2.37
**professional organizations**	2.21	2.19	2.53	2.35	2.32
**Researchers**	2.11	2.19	2.55	2.37	2.31
**regional government**	2.20	2.22	2.40	2.39	2.30
**local government**	2.28	2.22	2.43	2.39	2.33
**central government**	2.21	2.24	2.49	2.44	2.34
**EU**	2.32	2.29	2.51	2.44	2.39
**Average**	2.22	2.23	2.50	2.40	2.34

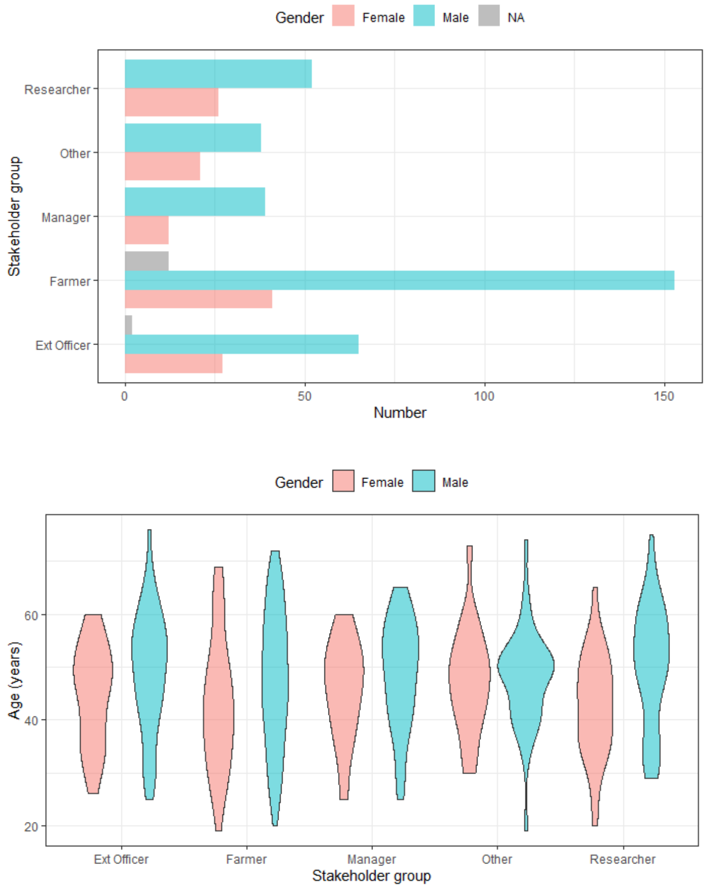

Figure S1. Participation plots according to profiles, gender, and age.
