# Supplementary material for: Assessing the vulnerability of mountain value chains to environmental and social drivers in Europe: A land-use and stakeholder-based approach
Source: Ambio. 2025 Mar 26;54(8):1386–403. doi: 10.1007/s13280-025-02153-5 (PMC12214118; doi:10.1007/s13280-025-02153-5)
Supplement: Supplementary file 2 — Supplementary file2 (PDF 590 KB) [file 13280_2025_2153_MOESM2_ESM.pdf]

Supplementary Material 2. Vulnerability Matrix per Region

Assessing the vulnerability of mountain value chains to environmental and social drivers in Europe: a land-use and stakeholder-based approach

Pablo González-Moreno[1,2*], Emilia Schmitt[3], Javier Moreno-Ortiz[3], Teresa Pinto-Correia[4], Nuno Guiomar[5], MOVING Consortium[6], María Mar Delgado-Serrano[3]

In this section, the calculated vulnerability matrices are presented for the 23 regions based on the scores of trends, ranking, sensitivity, and adaptation mechanisms collected from stakeholders. For each region, we provide two matrices. The first matrix shows the trend; the sensitivity (sensit); the mean reduction capacity of adaptation mechanisms considering: all mechanisms (reductALL), only medium feasible mechanisms (reductMF) and only high feasible mechanisms (reductHF); the impact (combining trend and sensitivity) and the vulnerability considering the three reduction scenarios (vulnALL, vulnMF and vulnHF) for each driver of change. Further explanation about this matrix is provided in Figure 1. The second matrix shows the weighted average of the same scores across all drivers considering the importance given by the ranking scores.

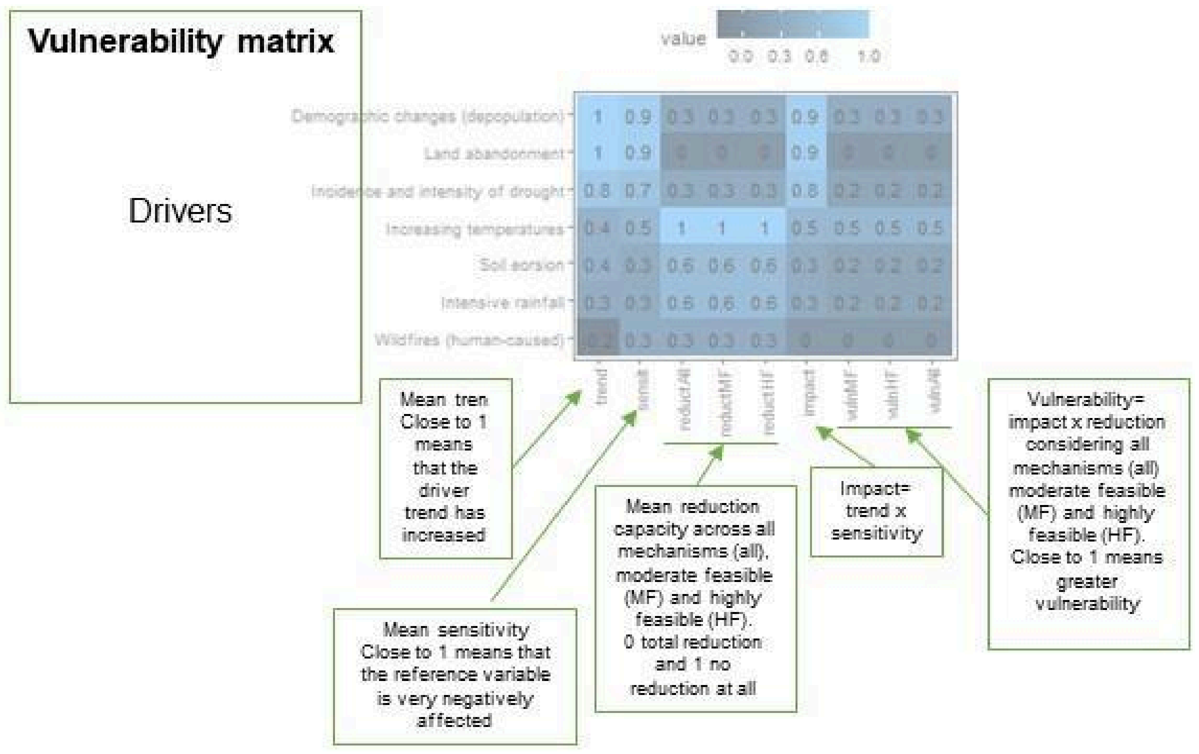

Figure 1 : vulnerability matrix explained

01 - Austrian Alps. Austria

Region: Austrian Alps MRL (region of Weiz)

Value Chain name: Lamb from the region of Weiz

Land-use system name: Extensive open range low land meadows and highland pastures

Name of the reference variable: Quality of pasture and linkage to the lamb production.

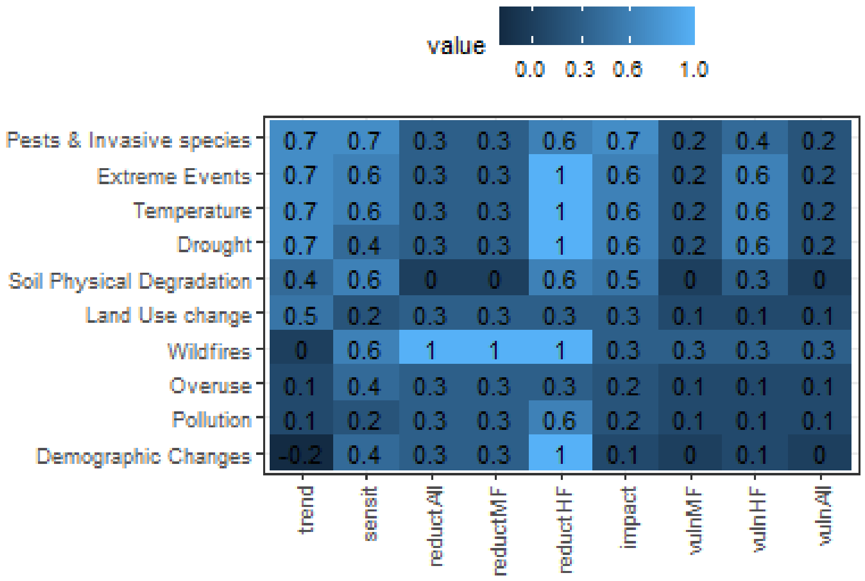

Figure 2. Summary table of values per driver for the Austrian Alps MRL (Austria)

The increased presence of wolf in the region seems to be of concern (pest & invasive species), that coupled with climatic drivers and soil physical degradation (partly due to climatic drivers), seem to be the drivers with the highest impact (Figure 2). Adaptive capacity is high in a scenario where all the proposed mechanisms are applied and of medium viability but decreases a lot in a scenario where only the most viable ones are applied (Table 1). This finding shows that adaptation to climate change and other difficulties is not an easy task, but it can be tackled even if this requires some effort.

Table 1 : Mean impact and vulnerability of the land use system for the Austria Alps MRL

Impact	VulnAll	vulnMF	vulnHF
**0.4**	0.1	0.1	0.3

02 - Stara Planina. Bulgaria

Region: Stara Planina MRL (Western Balkan Mountains)

Value Chain name: Public Goods from High Nature Value (HNV) farmland

Land-use system name: “Medium intensity / natural forest + Agro-silvo-pastoral system + Extensive open rangeland” – according to van Asselen and Verburg (2012) this might be broadly defined as an example of a “Mosaic (semi-) natural system” consisting of “Grassland and forest”

Name of the reference variable: “Farmland biodiversity” – specifically the “*presence of priority habitats of European significance that are dependent upon the continuation of traditional agriculture*”.

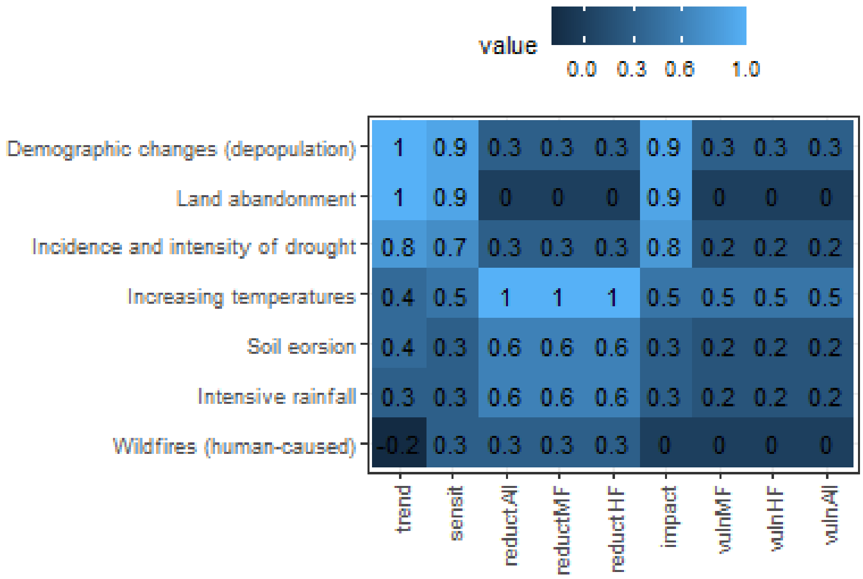

Figure 3 : Summary table of values per driver for Stara Planina MRL (Bulgaria)

Depopulation, land abandonment, and drought are the main factors, with a very high impact factor (0.9 and 0.8 out of 1; Figure 3). Only one of the top three factors is climatic. Already at medium values we see temperature increase, soil erosion and intensive rainfall (erosion to a large extent comes from intense rainfall), the climatic values are more appreciated. The overall impact of the system is quite high (Table 2), considering that it is a medium value, but it seems that there is also a high adaptive capacity of highly feasible mechanisms, with less potential to reduce the impact on the climate drivers.

Table 2. Mean impact and vulnerability of the land use system for the Stara Planina MRL (Bulgaria)

Impact	VulnAll	vulnMF	vulnHF
**0.6**	0.2	0.2	0.2

03 – Sumava - Cesky Les. Czechia

Region: Šumava - Český les MRL

Value Chain name: Beef Production

Land-use system name: Extensive open rangeland

Name of the reference variable: Quality pasture for cattle

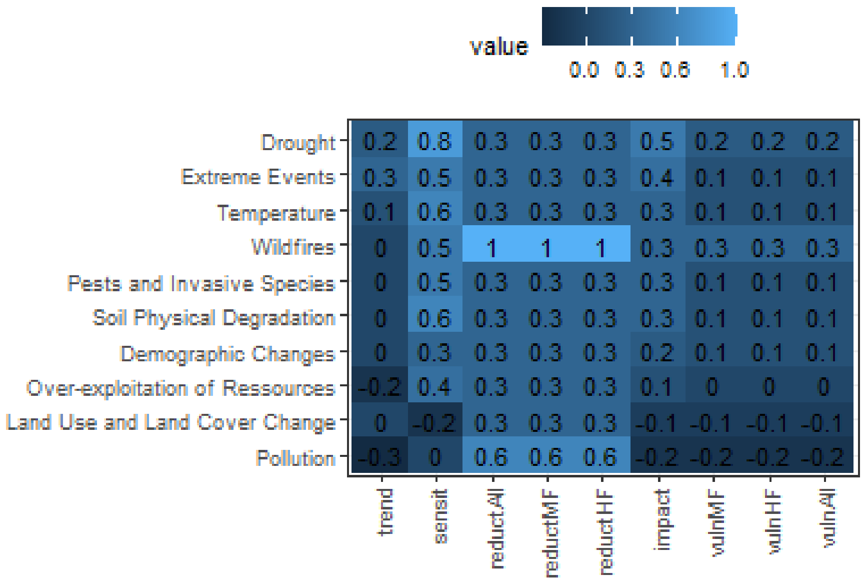

Figure 4 : Summary table of values per driver for Sumava- Cesky Les MRL (Czechia)

Although participants are aware of the high sensitivity that the reference variable (quality pasture for cattle) may have to some of the drivers such as drought, temperature, land degradation or extreme events, they do not currently see it as a potential problem, so the perceived vulnerability is low (Figure 4). Participants give more importance to how they are affected by regulations related to the National Park, Nature Protected Areas, or regulations of agricultural practices.

Table 3. Impact, adaptive capacity and vulnerability of the system for Sumava- Cesky Les MRL (Czechia)

impact	vulnAll	vulnMF	vulnHF
**0.2**	0.1	0.1	0.1

04 - Corsica. France

Region: Corsica MRL (Monte Renoso massif)

Value Chain name: PDO Chestnut flour

Land-use system name: Hight intensity plant forest (Agroforestry)

Name of the reference variable: Chestnut production.

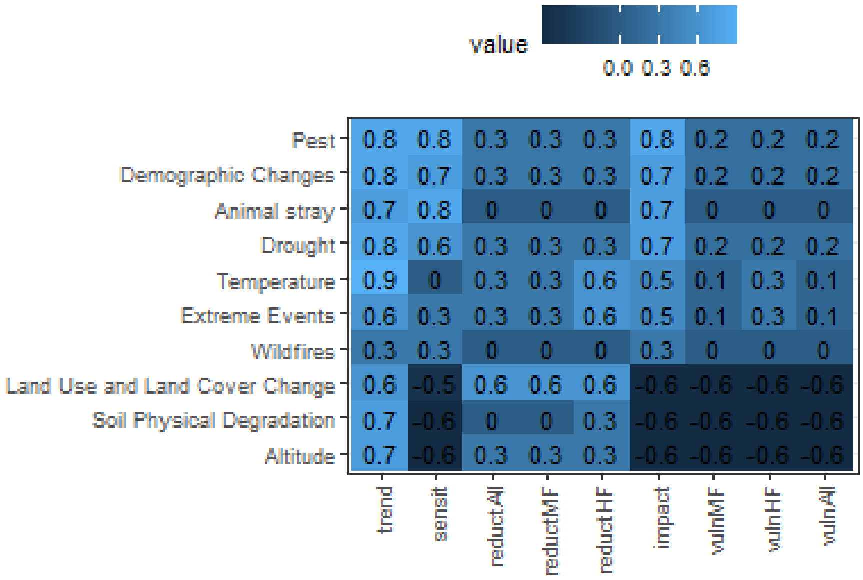

Figure 5 : Summary table of values per driver for Corsica MRL (France)

The drivers with higher impact perceived by participants were pests, demographic changes, animal stray and drought (Figure 5). Interestingly some drivers showed a high positive influence in the reference variable, which resulted in a rather low overall vulnerability (Table 4)

Table 4. Impact, adaptive capacity, and vulnerability of the system for Corsica MRL

impact	vulnAll	vulnMF	vulnHF
**0.3**	0	0	0

05 – Drôme Valley. France

Region: Drôme Valley MRL

Value Chain name: Sheep meat locally produced and valorised

Land-use system name: Extensive open pastures

Name of the reference variable: the pastoral resource: grasslands and shrubbery availability on the pasture areas

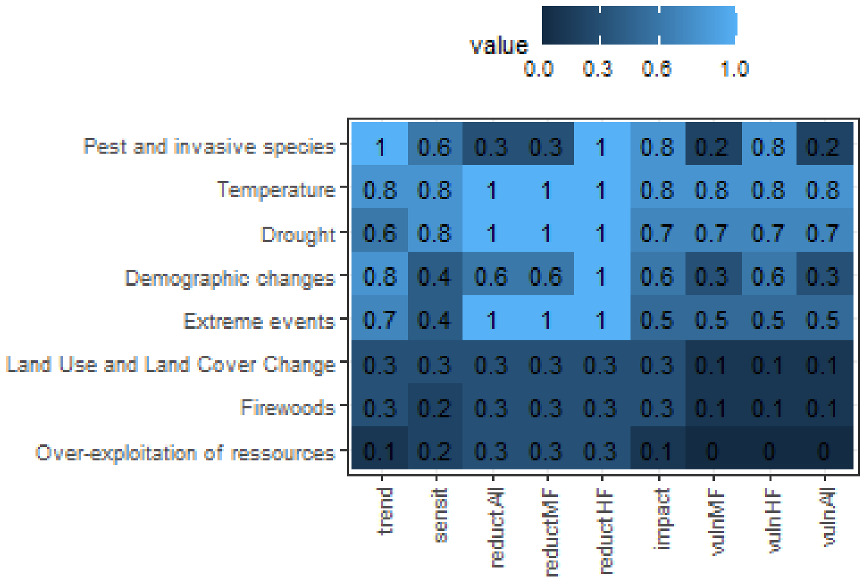

Figure 6 : Summary table of values per driver for Drôme Valley MRL (France)

The factors that are perceived to be most influential are invasive species (with the wolf issue), climatic drivers (temperature, drought and extreme events) and demographic changes (Figure 6). There is little perceived adaptive capacity to these drivers. In contrast, in other case studies we have found mechanisms that can also influence the reduction of the impact of climate drivers, it may be interesting to see if they could be applicable to the context of the Drôme Valley.

Table 5. Impact and vulnerability of the land use system for Drôme Valley MRL (France)

impact	vulnAll	vulnMF	vulnHF
**0.5**	0.4	0.4	0.5

06 - Crete. Greece

Region: Crete MRL (Rethymno)

Value Chain name: Central Rethymno Carob flour

Land-use system name: Agro-Silvo-Pastoral

Name of the reference variable: Carob Pod Production

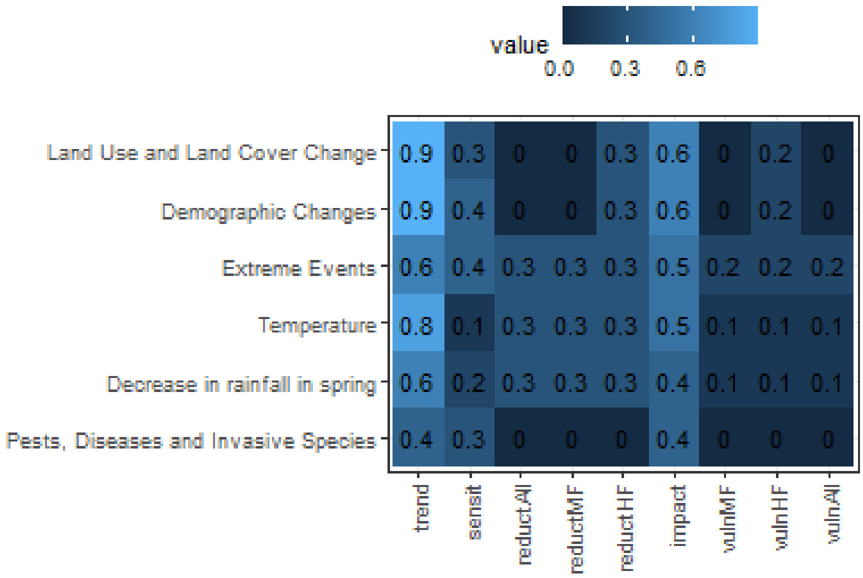

Figure 7 : Summary table of values per driver for Crete MRL (Greece)

A combination of climatic (extreme events, spring drought) and social drivers of change (depopulation, abandonment and change of use) can affect the reference variable in a negative way (Figure 7). Undoubtedly, avoiding the abandonment of carob crops or the change to olive groves is closely linked to giving greater value to carob products, something which, together with the application of adaptation mechanisms, could greatly reduce the vulnerability of the system.

Table 6. Impact and vulnerability of the land use system for Crete MRL (Greece)

impact	vulnAll	vulnMF	vulnHF
0.5	0.1	0.1	0.1

07 - Transdanubian Mountains. Hungary

Region: Transdanubian Mountains MRL

Value Chain name: Agroecological knowledge - Cold Mountain Shelter knowledge economy

Land-use system name: Mosaic land use system

Name of the reference variable: the quantity of healthy food produced through mosaic land use in agro-ecological farming

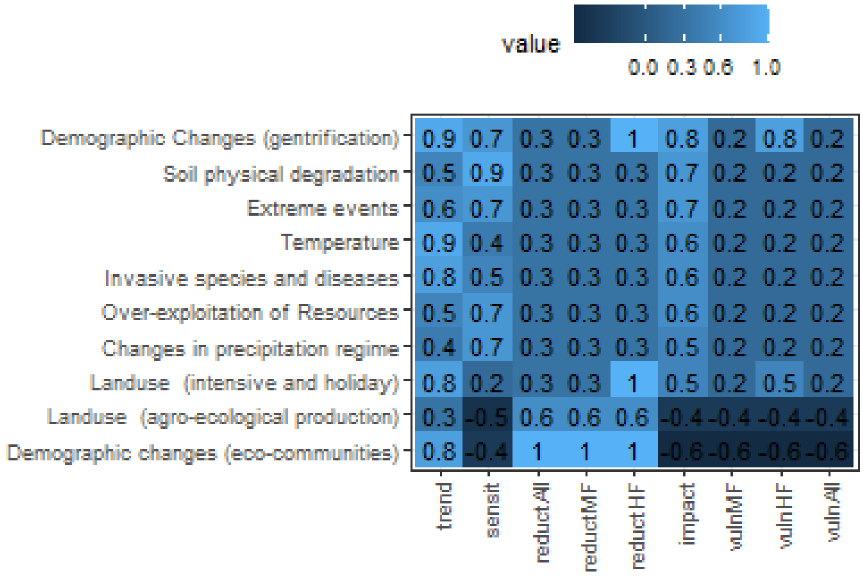

Figure 8 : Summary table of values per driver for Transdanubian MRL (Hungary)

The combination of drivers with high negative impact (positive score) with two drivers of change that have a positive impact on the reference variable (negative scores), greatly reduces overall impact (Table 7). Despite this, we cannot downplay the high impact values of the other drivers, especially those related to gentrification, land degradation, and climate drivers (Figure 8). Adaptation mechanisms have a high feasibility to be applied, although the overall adaptive capacity to these drivers is medium (not complete). Here again, it is important to have drivers of change that improve the situation, in order to compensate for the above.

Table 7. Impact and vulnerability of the land use system for Transdanubian MRL (Hungary)

impact	vulnAll	vulnMF	vulnHF
**0.4**	0.1	0.1	0.1

08 - Central Apennines. Italy

Region: Central Apennines MRL

Value Chain name: Alto Molise dairy production. Caciocavallo cheese.

Land-use system name: Agro-silvo-pastoral system (largely relevant for the VC).

Name of the reference variable: Permanent grasslands and meadows.

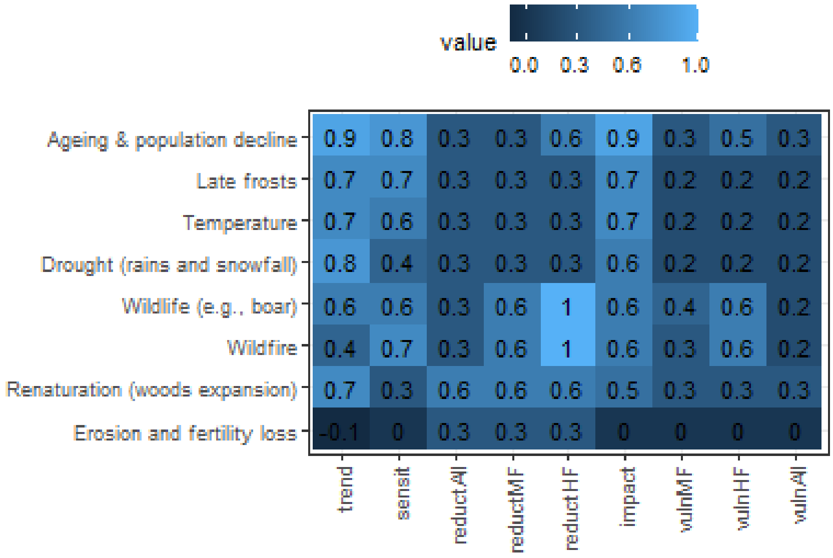

Figure 9 : Summary table of values per driver for Central Apennines MRL (Italy)

As in many rural areas ageing and population decline is one of the major challenges, which require adaptation mechanisms. In this case, the perception of the participants is that they are not very optimistic about the feasibility and full effectiveness of the mechanisms. Climate drivers (drought, temperature, and late frost), wild animals such as boar, and fires are other threats. Interestingly, and contrary to other cases, a greater feasibility is seen in the application of measures against climate drivers than in drivers of change with a social component.

Table 8. Impact and vulnerability of the land use system for Central Apennines MRL (Italy)

Impact	VulnAll	vulnMF	vulnHF
**0.6**	0.2	0.2	0.3

09 - Eastern Alps. Italy

Region: Eastern Alps MRL

Value Chain name: Trento Doc Wine - Mountain viticulture in Alto Trentino

Land-use system name: Permanent cropland

Name of the reference variable: vineyard productivity and grape quality

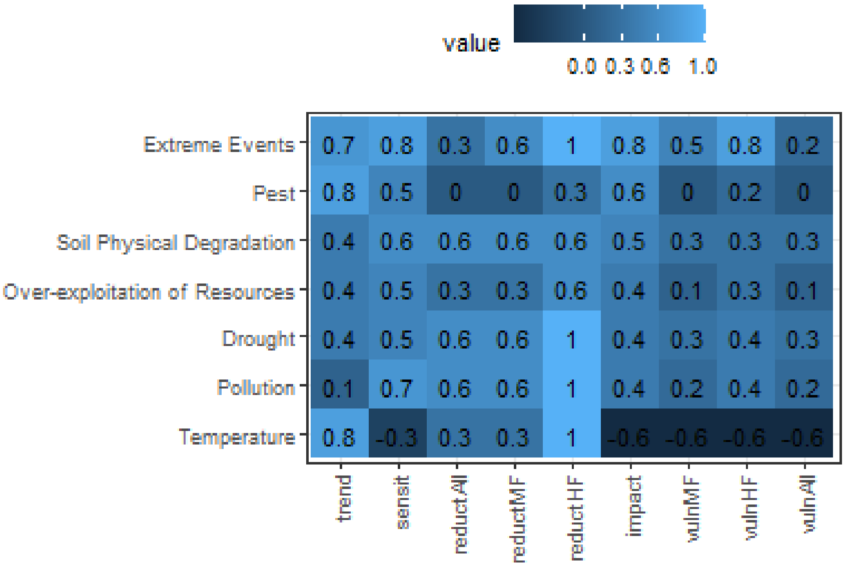

Figure 10 : Summary table of values per driver for Eastern Alps MRL (Italy)

Extreme events, in particular spring frosts and heavy rainfall, is the factor with the greatest impact on vineyard productivity and grape quality (Figure 10). In fact, heavy rainfall also causes soil erosion and compaction (soil physical degradation). Adaptive mechanisms for these drivers have a medium/low viability and impact reduction capacity. Similar is the case for drought and pollution, although with a lower impact value. Other factors, such as pests and over-exploitation of resources, are perceived as more controllable. In contrast, the positive impact that temperature can have on high mountain viticulture stands out.

Table 9. Impact and vulnerability of the land use system for Eastern Alps MRL (Italy)

Impact	VulnAll	vulnMF	vulnHF
[redacted]	[redacted]	[redacted]	[redacted]

10 - Northern Apennines. Italy

Region: Northern Apennines MRL

Value Chain name: Chestnut flour

Land-use system name: High intensity plant forest / Agroforestry

Name of the reference variable: “chestnuts tree varieties” that influence the high quality of the chestnut flour but also the annual production (quantity) of chestnuts.

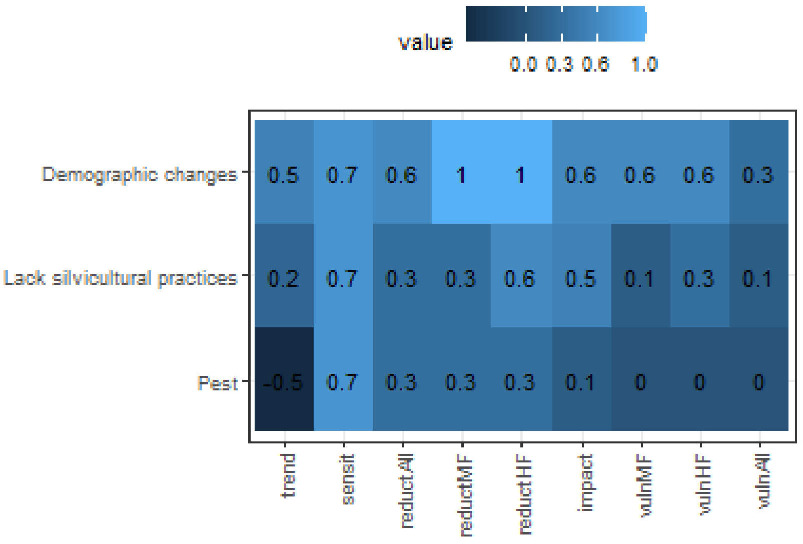

Figure 11 : Summary table of values per driver for Northern Apennines MRL (Italy)

In this case study, the vulnerability of the system has been assessed taking into account only three drivers, considered to be the most important (Figure 11). The two main drivers are linked to each other, as it is the same social dynamics that cause both depopulation and the loss of knowledge and practices. The perception of adaptive capacity is quite negative, with little or no capacity to reduce impacts, and few mechanisms with high feasibility. In the case of pests, the trend in recent years has already been positive and there is a potential to reduce the average impact, which makes expectations better.

Table 10. Impact and vulnerability of the land use system for Northern Apennines MRL (Italy)

Impact	VulnAll	vulnMF	vulnHF
[redacted]	[redacted]	[redacted]	[redacted]

11 - Maleshevski mountains. North Macedonia

Region: Maleshevski mountains MRL

Value Chain name: Rural tourism

Land-use system name: Mosaic/landscape of Maleshevski region (predominant medium intensity /natural forest)

Name of the reference variable: Landscape of Maleshevija.

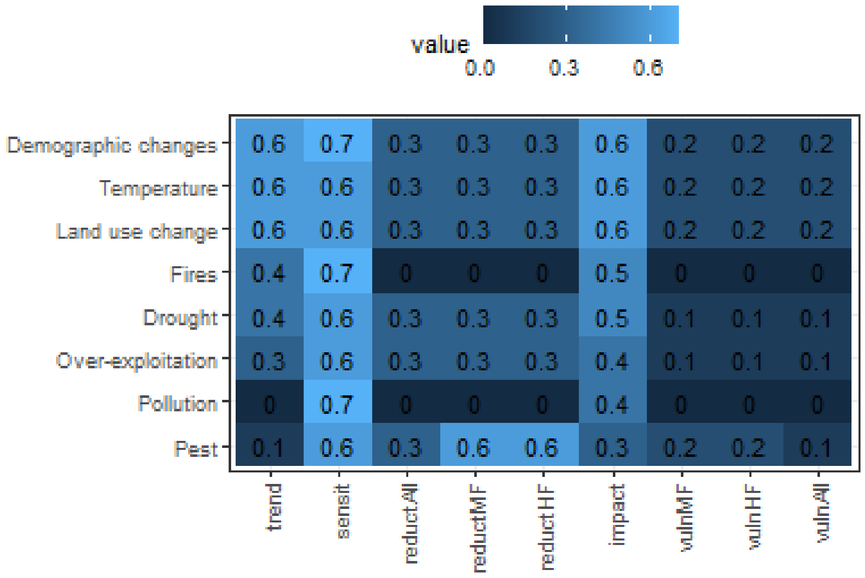

Figure 12 : Summary table of values per driver for Maleshevsky mountains MRL (North Macedonia)

Participants perceived a medium impact (between 0.4 and 0.6) of most drivers (Figure 12). Optimism about adaptive capacity mechanisms stands out positively, as a high feasibility and a medium reduction capacity is perceived, which would greatly improve the situation, in the face of an impact of these drivers of change. For pests, on the other hand, it seems that this is not currently an important factor, but it would potentially have a large impact and is still perceived to have less coping capacity than the other drivers.

Table 11. Impact and vulnerability of the land use system for Maleshevsky mountains MRL (North Macedonia)

impact	vulnAll	vulnMF	vulnHF
**0.5**	0.1	0.1	0.1

12 - Cordilheira Central. Portugal

Region: Cordilheira Central MRL (Serra da Estrela)

Value Chain name: Serra da Estrela PDO Cheese

Land-use system name: Extensive Open Rangeland (3), more specifically, highland and lowland pastures

Name of the reference variable: Pasture area

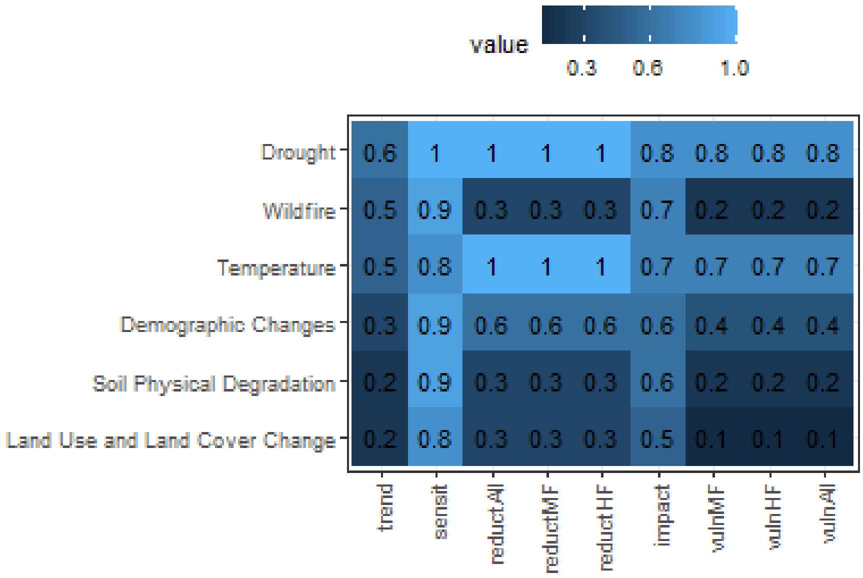

Figure 13 : Summary table of values per driver for Cordilheira Central MRL (Portugal)

In this case study, the sensitivity of each of the drivers is so high that the system is potentially highly threatened, although this sensitivity is compensated by a trend for some drivers to be lower (Figure 13). The adaptive capacity for the climatic drivers in this case does not seem to be effective, although it does seem to be medium effective and with high viability for the rest. The final result is a relatively high impact for the system (Table 12), which translates into a medium vulnerability, due to the decreasing capacity of the adaptation mechanisms.

Table 12. Impact and vulnerability of the land use system for Cordillheira Central MRL (Portugal)

Impact	VulnAll	vulnMF	vulnHF
**0.6**	0.4	0.4	0.4

13 - Maciço Noroeste. Portugal

Region: Maciço Noroeste MRL (Douro Superior)

Value Chain name: Dour Wine Value Chain

Land-use system name: Permanent cropland

Name of the reference variable: The vineyard productivity and grape quality

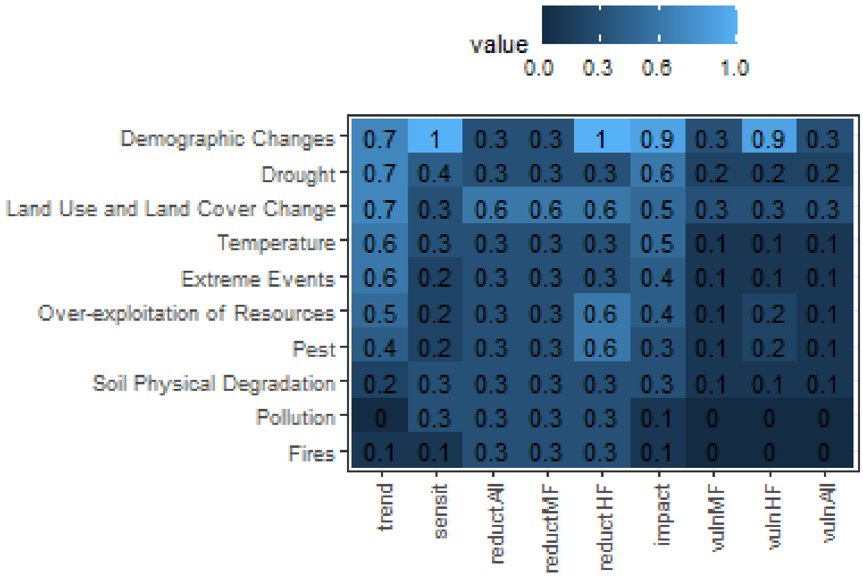

Figure 14 : Summary table of values per driver for Maciço Noroeste MRL (Portugal)

The high impact value for demographic changes stands out as one of the highest obtained in the case study, far ahead of the second one, which would be drought (Figure 14). The adaptive capacity mechanisms generally have a medium reduction capacity and high viability, except for demographic changes, overexploitation of resources and pest, where the mechanisms with the highest viability have a lower impact reduction capacity which means that in a scenario where only the most viable mechanisms are applied, the vulnerability would be 0.2 (Table 13), in contrast to 0.1 if all the mechanisms or the mechanisms with medium viability were applied.

Table 13. Impact and vulnerability of the land use system for Maciço Noroeste MRL (Portugal)

Impact	VulnAll	vulnMF	vulnHF
**0.4**	0.1	0.1	0.2

14 - Southern Romanian Carpathian Mountains. Romania

Region: Southern Romanian Carpathian Mountains MRL (Piatra Craiului National Park)

Value Chain name: Certified Ecotourism

Land-use system name: Mosaic landscape with forest and extensive semi-natural grassland

Name of the reference variable: “landscape composition” was considered more subjectively as the basis of the “highly appreciated natural beauty of the area”

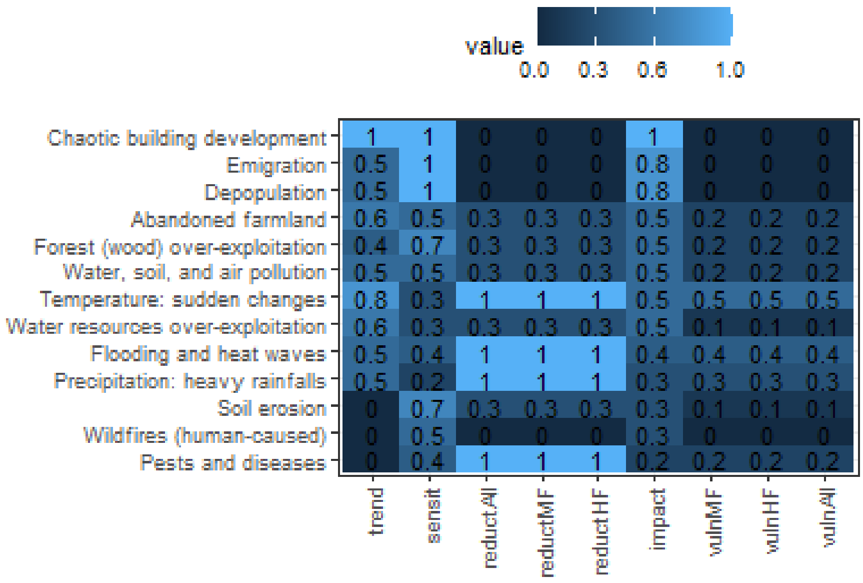

Figure 15 : Summary table of values per driver for Southern Romanian Carpathians MRL

Chaotic building development, emigration and depopulation are drivers of change showing a very large potential impact on the system, although in turn, there seems to be a full capacity to reduce this impact with mechanisms with high feasibility (Figure 15). The rest of the drivers, both climate and social components, show a medium impact, which can be reduced in a medium way in the social components. On the other hand, the selected mechanisms do not have the capacity to reduce the climate drivers (Figure 15). In general, there is a high capacity to reduce the impact on the system, from an impact of 0.6 to 0.1 across all adaptation scenarios (Table 14).

Table 14. Impact and vulnerability of the land use system for Southern Romanian Carpathians MRL

Impact	VulnAll	vulnMF	vulnHF
**0.6**	0.1	0.1	0.1

15 - Dinaric Mountains. Serbia

Region: Dinaric Mountains MRL. (Sjenica – Pester plateau)

Value Chain name: Sjenica lamb meat (PDO)

Land-use system name: Extensive open rangeland /Mosaic cropland (extensive) and grassland with few livestock/

Name of the reference variable: Productivity and quality of the pastures on the highlands.

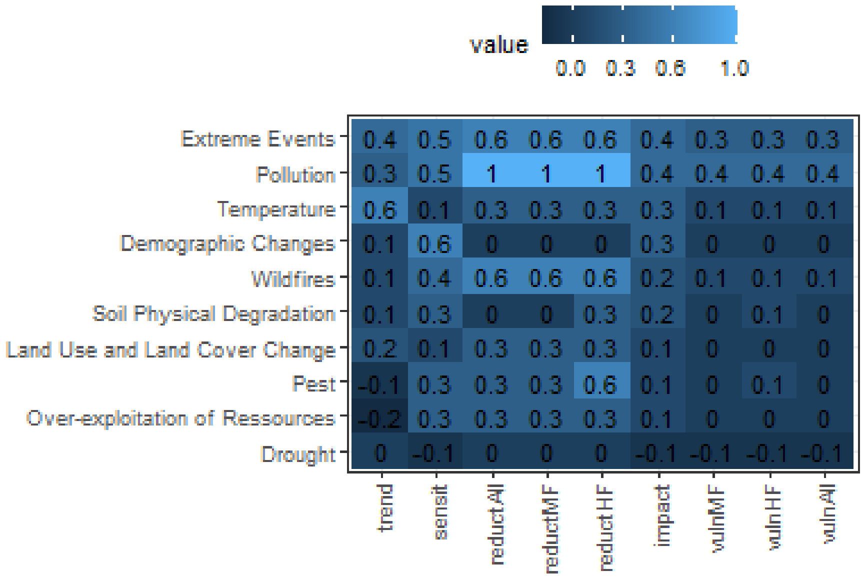

Figure 16 : Summary table of values per driver for Dinaric Mountains MRL (Serbia)

At a general overview, it can be observed that very high impact values are not perceived, with only extreme events, pollution and demographic changes standing out, due to the fact that sensitivity in general is medium and is accompanied by medium/low trends (Figure 16). In addition, for some drivers such as demographic changes, soil physical degradation has a full impact reduction potential capacity, which also contributes to a low overall vulnerability of the system. On the other hand, the application of adaptation mechanisms would not be sufficient for extreme events and pollution to significantly reduce their impact, with possible consequences derived from this.

Table 15. Impact and vulnerability of the land use system for Dinaric Mountains MRL (Serbia)

Impact	VulnAll	vulnMF	vulnHF
**0.2**	0.1	0.1	0.1

16 - Slovak Carpathian Mountains. Slovakia

Study case: Slovak Carpathians MRL

Value Chain name: Bio-honey

Land-use system name: extensive grasslands (pastures and meadows) and forest

Name of the reference variable: Diversity of pollen- and nectar – producing plants and honeydew from trees.

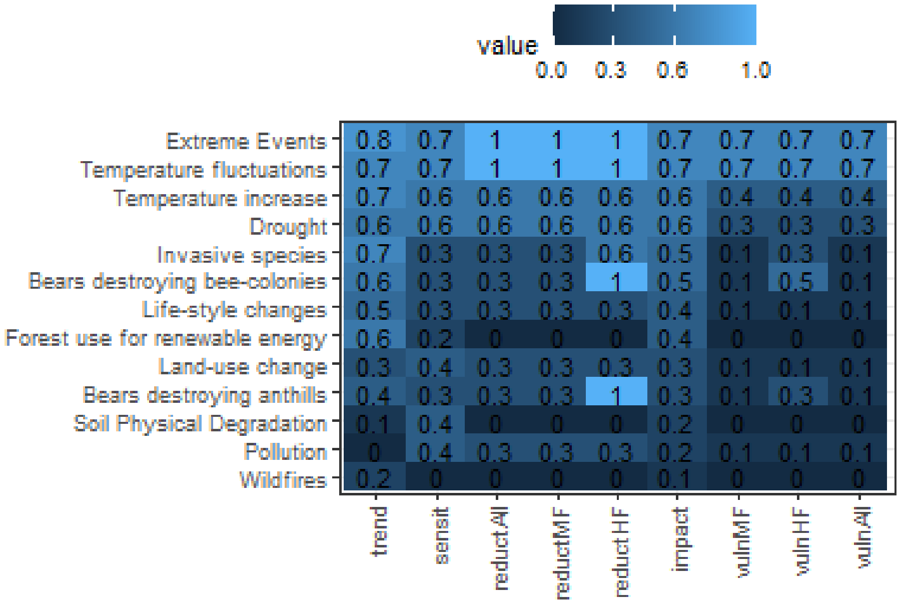

Figure 17 : Summary table of values per driver for Slovak Carpahtian mountains MRL (Czech Republic)

The drivers with the greatest impact, are the climatic drivers (Extreme events, temperature increase and fluctuation and drought), for which there is none or a low potential to reduce the impact of the drivers (Figure 17), which could be worrying for this value chain. For the rest of the drivers, with average impact values, a greater capacity for adaptation is perceived.

Table 16. Impact and vulnerability of the land use system for Slovak Carpahtian mountains MRL

Impact	VulnAll	vulnMF	vulnHF
**0.5**	0.2	0.2	0.3

17 - Betic Systems. Spain

Region: Betic Systems MRL (Subbetica Cordobesa)

Value Chain name: Organic Mountain olive oil

Land-use system name: Betic Systems

Name of the reference variable: The productivity of organic olives (in terms of quantity and quality)

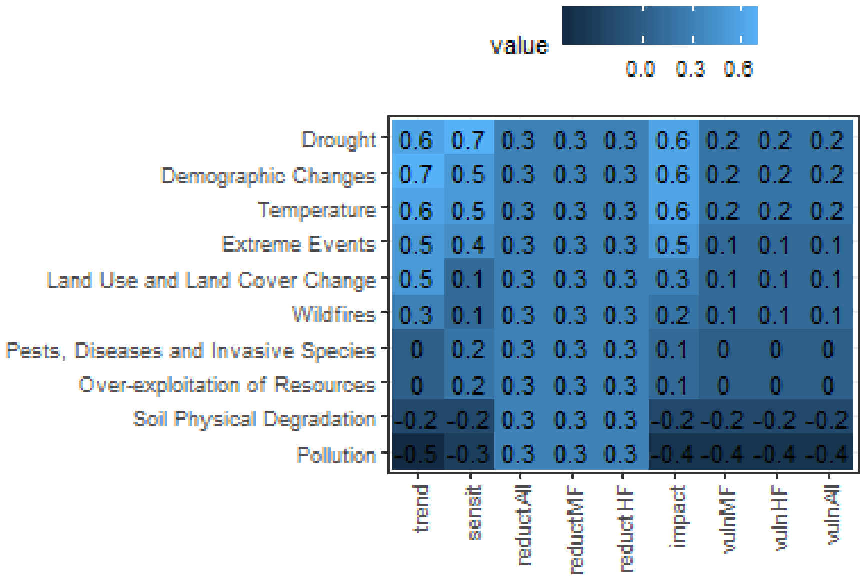

Figure 18 : Summary table of values per driver for Betic systems MRL (Spain)

Climate drivers (drought, temperature and extreme events) and demographic changes (depopulation) are the drivers with the greatest potential impact on olive productivity (Figure 18). It should be noted that the average potential impact reduction capacity and high viability of the adaptation mechanisms is high. The negative values in sensitivity and trend for the drivers of physical soil degradation and contamination are due to the improvement of the soil and the absence of contamination due to the change from conventional to organic production.

Table 17. Impact and vulnerability of the land use system for Betic systems MRL (Spain)

impact	vulnAll	vulnMF	vulnHF
**0.3**	0.1	0.1	0.1

18 - Sierra Morena. Spain

Region: Sierra Morena MRL (Pozoblanco, Villanueva de Cordoba, and Cardeña)

Value Chain name: Los Pedroches PDO Iberian Ham

Land-use system name: Agro-silvo-pastoral system

Name of the reference variable: Production of grass and acorns

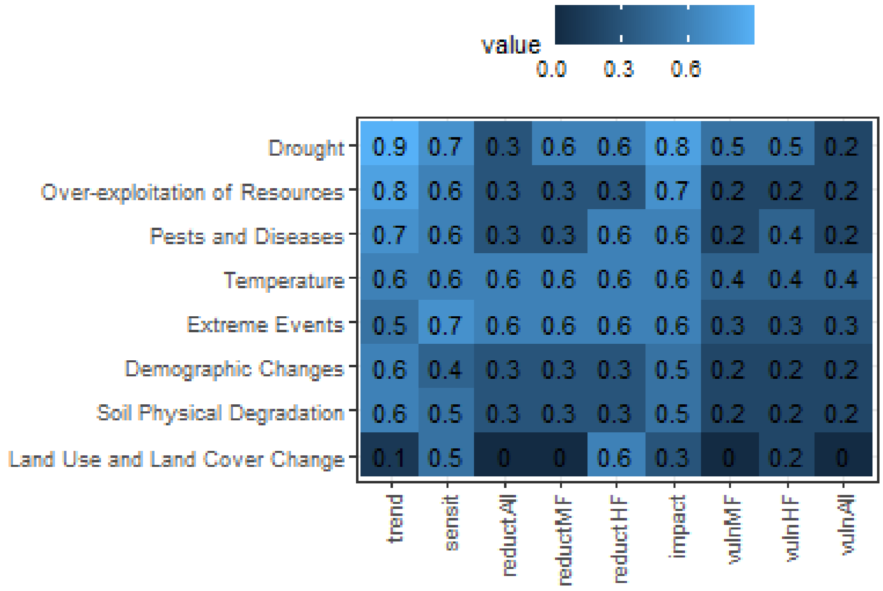

Figure 19 : Summary table of values per driver for Sierra Morena MRL (Spain)

Impact values are high for most drivers ranging from 0.5 to 0.8 (Figure 19). Drought and overexploitation (livestock load) are the biggest threats, closely followed by pests ("*Phytophthora cinnamomi*"), temperature and extreme events. Adaptive capacity is low for the climate drivers, and medium-low for the rest, depending on the feasibility of the measures. Strong support is needed to ensure that most adaptive mechanisms can be implemented.

Table 18. Impact and vulnerability of the land use system for Sierra Morena MRL (Spain)

impact	vulnAll	vulnMF	vulnHF
**0.6**	0.2	0.3	0.3

19 - Spanish Pyrenees. Spain

Region: Spanish Pyrenees MRL

Value Chain name: Mountain Wine Value Chain (Spanish Vignerons from pre-Pyrenean mountains)

Land-use system name: permanent cropland

Name of the reference variable: The vineyard productivity and grape quality

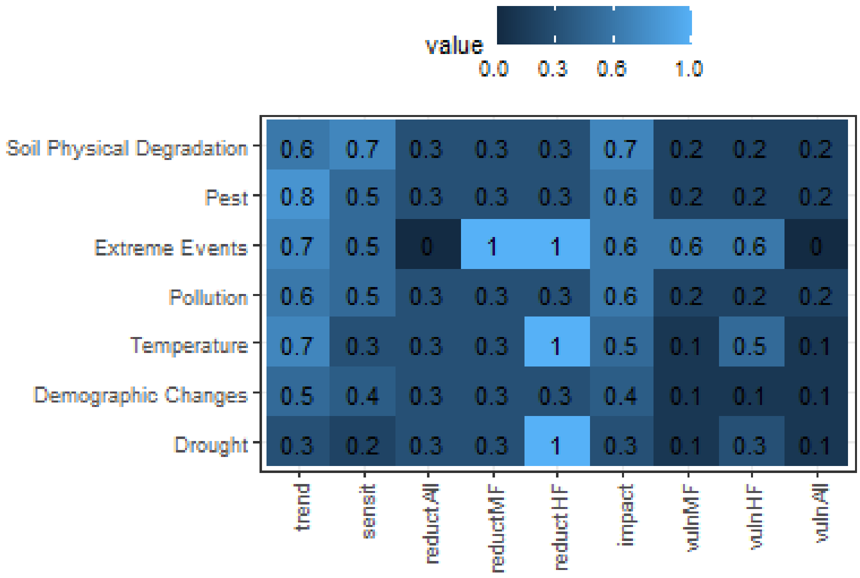

Figure 20 : Summary table of values per driver for Spanish Pyrenees MRL (Spain)

The drivers of change with the greatest impact are physical land degradation and pollution due to poor management, pests, climatic drivers (extreme events, temperature and drought), and demographic changes (Figure 20). Perhaps this last driver is underestimated, as some of the participants lived outside the MRL. Less adaptive capacity is observed for climatic drivers, for which the feasibility of adaptation mechanisms is lower. In contrast, for the other drivers, high feasibility and medium impact reduction potential of the adaptation mechanisms is observed. This difference in feasibility means that vulnerability changes depending on the feasibility scenario.

Table 19. Impact and vulnerability of the land use system for Spanish Pyrenees MRL

impact	vulnAll	vulnMF	vulnHF
**0.5**	0.1	0.2	0.3

20 – Swiss Alps. Switzerland

Region: Swiss Alps MRL

Value Chain name: Mountain Grain value chain (Grisons Mountain Cereals)

Land-use system name: Cropland extensive

Name of the reference variable: “Cereal yields” in kg/ha

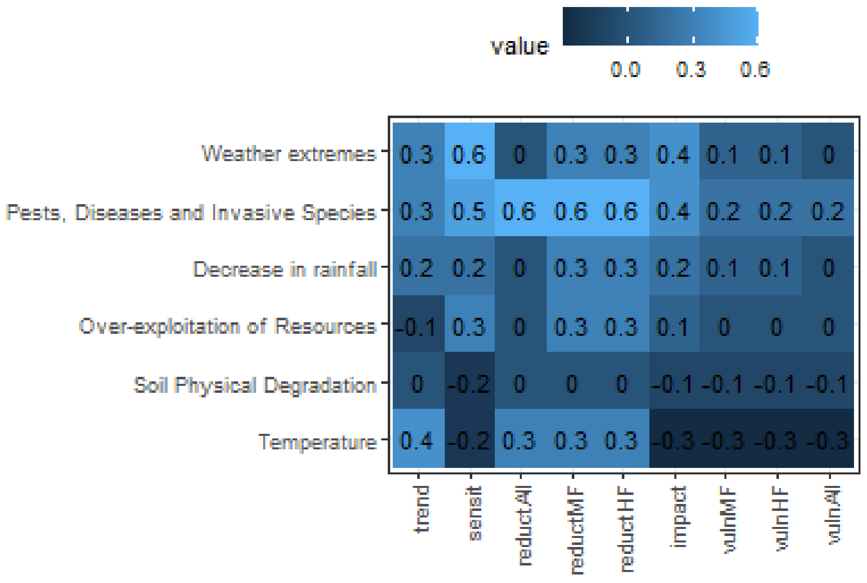

Figure 21 : Summary table of values per driver for Swiss Alps MRL (Switzerland)

Two drivers stand out with a medium impact, weather extremes and "weeds" (Pests, diseases, and invasive species) (Figure 21). Two drivers with a positive impact also stand out. Temperature, because an increase in temperature in the mountains will improve crop productivity, and physical soil degradation, because good management of cereals can improve the soil. In general, adaptive capacity mechanisms have a medium to full impact reduction potential, with high feasibility, except in the case of weeds, where the reduction capacity is low.

Table 20. Impact and vulnerability of the land use system for Swiss Alps MRL

Impact	VulnAll	vulnMF	vulnHF
0.2	0	0.1	0.1

21 - Swiss Jura. Switzerland

Region: Swiss Jura MRL

Value Chain name: Tête de Moine PDO

Land-use system name: Extensive open rangeland

Name of the reference variable: Annual production of grass and fodder expressed in terms of quantity and species diversity.

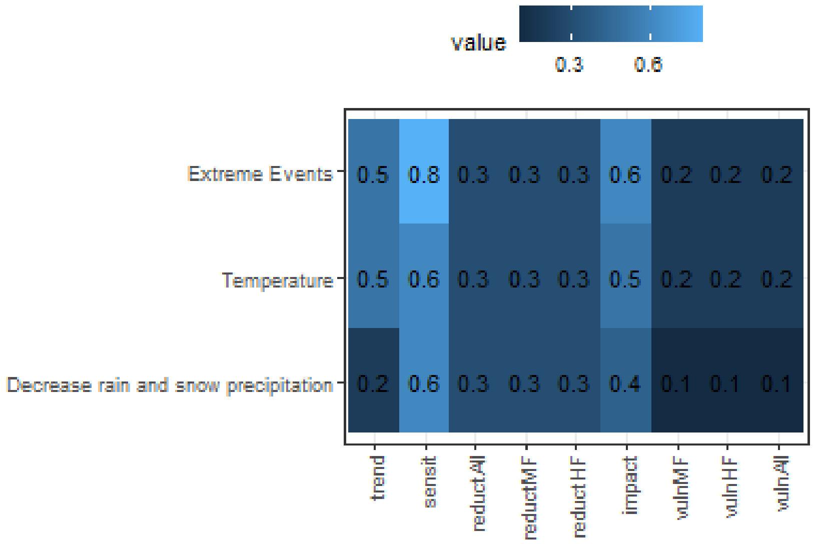

Figure 22 : Summary table of values per driver for Swiss Jura MRL (Switzerland)

In this case study, to calculate the vulnerability, only the three most relevant drivers were selected according to the ranking scores. Those drivers were extreme events, temperature and decrease rain and snow precipitation. The impact of the three selected drivers is medium-high (0.4-0.6) (Figure 22), but there are mechanisms with high feasibility and moderate impact reduction capacity, which if applied would reduce the vulnerability of the system.

Table 21. Impact and vulnerability of the land use system for Swiss Jura MRL

Impact	VulnAll	vulnMF	vulnHF
0.5	0.2	0.2	0.2

22 – Beydaglari. Turkey

Region: Beydaglari MRL

Value Chain name: Greenhouse Tomato

Land-use system name: Intensive annual crop / irrigated

Name of the reference variable: Greenhouse Tomato Production

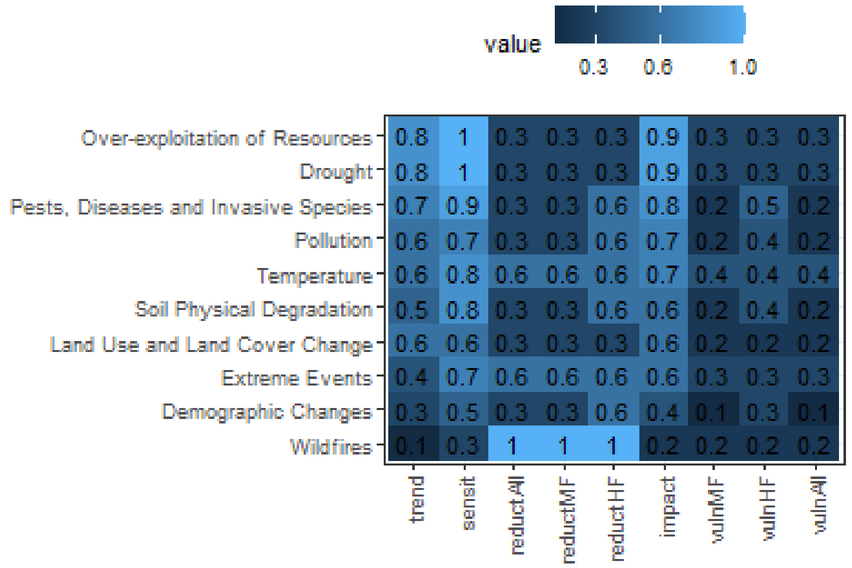

Figure 23 : Summary table of values per driver for Beydaglary MRL (Turkey)

There is a high impact risk for the coming years for many drivers, warning of the need for urgent action. Fortunately, there appear to be mechanisms with high feasibility that could be applied to reduce vulnerability. Although it will require very strong support, to be able to implement mechanisms with medium and low feasibility in some cases, as well as creativity to be able to develop new ones. Issues related to water scarcity, temperature rise, disease, pollution, and soil degradation seem to need special attention.

Table 22. Impact and vulnerability of the land use system for Beydaglary MRL (Turkey)

Impact	VulnAll	vulnMF	vulnHF
0.7	0.2	0.2	0.3

23 –Highlands and Islands. United Kingdom – Scotland

Region: Speyside MRL

Value chain name: Speyside Malt Whisky

Land-use system name: Extensive Rangeland

Name of the reference variable: Water Quantity

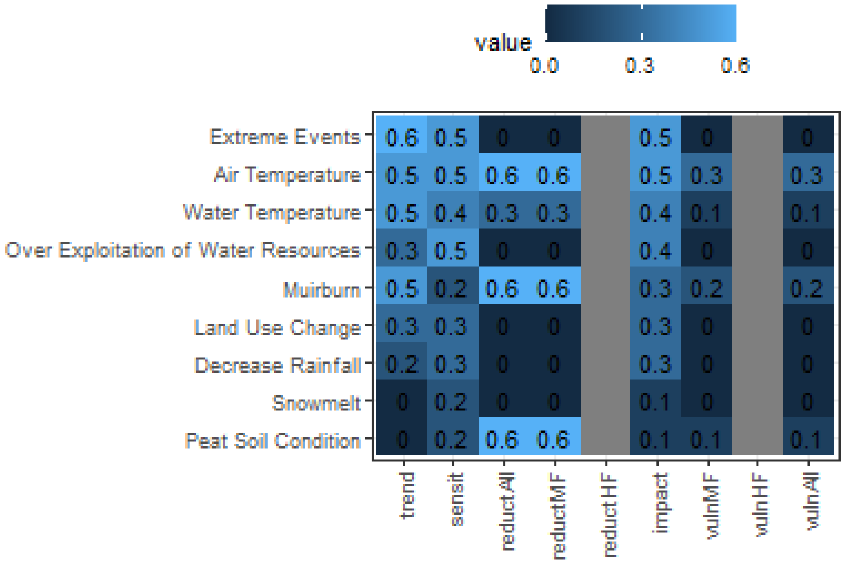

Figure 24 Summary table of values per driver for Speyside MRL (United Kingdom)

Overall, the vulnerability of the system is not very high (Table 23). This pattern results from some drivers that have a low impact and compensate for other drivers with relatively high impact values such as extreme events, air and water temperature and over exploitation of water resources (Figure 24). When calculating the vulnerability, we observe that for some drivers a complete reduction of impact is possible. Likewise, there are no mechanisms with high feasibility (due to lack of economic viability), which will require a strong involvement of the administration in order to address these challenges.

Table 23 : . Impact and vulnerability of the land use system for Speyside MRL (UK)

Impact	VulnAll	vulnMF	vulnHF
**0.3**	0.1	0.1	NA
